# Polyketide Starter and Extender Units Serve as Regulatory Ligands to Coordinate the Biosynthesis of Antibiotics in Actinomycetes

**DOI:** 10.1128/mBio.02298-21

**Published:** 2021-09-28

**Authors:** Panpan Wu, Ketao Chen, Bowen Li, Yanni Zhang, Hang Wu, Yuhong Chen, Shaohua Ren, Sabir Khan, Lixin Zhang, Buchang Zhang

**Affiliations:** a Institute of Physical Science and Information Technology, School of Chemistry and Chemical Engineering, School of Life Sciences, Anhui University, Hefei, China; b State Key Laboratory of Bioreactor Engineering, East China University of Science and Technology, Shanghai, China; McMaster University

**Keywords:** actinomycetes, *Saccharopolyspora erythraea*, polyketide, regulatory ligands, AcrT

## Abstract

Polyketides are one of the largest categories of secondary metabolites, and their biosynthesis is initiated by polyketide synthases (PKSs) using coenzyme A esters of short fatty acids (acyl-CoAs) as starter and extender units. In this study, we discover a universal regulatory mechanism in which the starter and extender units, beyond direct precursors of polyketides, function as ligands to coordinate the biosynthesis of antibiotics in actinomycetes. A novel acyl-CoA responsive TetR-like regulator (AcrT) is identified in an erythromycin-producing strain of Saccharopolyspora erythraea. AcrT shows the highest binding affinity to the promoter of the PKS-encoding gene *eryAI* in the DNA affinity capture assay (DACA) and directly represses the biosynthesis of erythromycin. Propionyl-CoA (P-CoA) and methylmalonyl-CoA (MM-CoA) as the starter and extender units for erythromycin biosynthesis can serve as the ligands to release AcrT from P*_eryAI_*, resulting in an improved erythromycin yield. Intriguingly, anabolic pathways of the two acyl-CoAs are also suppressed by AcrT through inhibition of the transcription of acetyl-CoA (A-CoA) and P-CoA carboxylase genes and stimulation of the transcription of citrate synthase genes, which is beneficial to bacterial growth. As P-CoA and MM-CoA accumulate, they act as ligands in turn to release AcrT from those targets, resulting in a redistribution of more A-CoA to P-CoA and MM-CoA against citrate. Furthermore, based on analyses of AcrT homologs in Streptomyces avermitilis and Streptomyces coelicolor, it is believed that polyketide starter and extender units have a prevalent, crucial role as ligands in modulating antibiotic biosynthesis in actinomycetes.

## INTRODUCTION

Actinomycetes are important sources of many bioactive secondary metabolites, including antibiotics, insecticides, cholesterol-lowering agents, anticancer drugs, and immunosuppressants (1). They are intelligent microbes that balance the biosynthesis of different metabolites in cells through precise modulation (1). Secondary metabolites are produced by actinomycetes using primary metabolites as precursors (2). The transcription of antibiotic biosynthetic gene clusters is generally dependent on cluster-situated regulators (CSRs) or global/pleiotropic regulators (2). Both the onset and yield of antibiotic biosynthesis are modulated by different regulatory factors, some of which respond to diverse physiological or environmental signals (3). These specialized metabolites, such as hormone-like autoregulators (4–7), antibiotics themselves, or their biosynthetic intermediates (8–14), modulate the DNA-binding activity of these allosteric regulators (3). However, due to the difficulties in identifying novel ligands and characterizing their receptors, the underlying mechanisms of ligand-mediated regulation remain poorly understood.

Polyketides encompass a large class of secondary metabolites with various structures and biological activities and represent a significant source of new drugs (15). During polyketide biosynthesis, multiple types of coenzyme A esters of short fatty acids (acyl-CoAs), as original building blocks, are condensed by multifunctional enzymes called polyketide synthases (PKSs) (16, 17). Three types of PKSs (type I, II, and III) have been reported and rationally engineered via synthetic biology or metabolic engineering approaches to design new polyketides or improve polyketide yield (18–20). Actinomycetes are well-known producers of type I and II polyketides (21, 22). In addition to polyketide starter and extender units, acyl-CoAs also serve as acyl donors for protein posttranslational modification to modulate various metabolic processes in actinomycetes (23–25). Although a recent report demonstrated the functional relationship between acylation regulation and secondary metabolism (26), the acyl-CoA-mediated signal regulation in antibiotic biosynthesis has remained elusive.

Saccharopolyspora erythraea (*Sac. erythraea*), as the model bacterium, has been extensively utilized to investigate polyketide biosynthesis for chemical diversification and titer improvement (19). Erythromycin A (Er-A), a typical type I polyketide, is assembled from one molecule of propionyl-CoA (P-CoA) starter unit and six molecules of (*S*)-methylmalonyl-CoA [(*S*)-MM-CoA] extender unit to build the macrolactone backbone 6-deoxyerythronolide B, which is further decorated by tailoring reactions including hydroxylation, glycosylation, and methylation (27). In *Sac. erythraea*, the acetyl-CoA (A-CoA) carboxylase (ACC) pathway (carboxylating A-CoA to malonyl-CoA [M-CoA]) plays a key role in the intracellular supply of P-CoA, while MM-CoA can be synthesized via the P-CoA carboxylase (PCC) pathway (carboxylating P-CoA to MM-CoA) and the MM-CoA mutase (MCM) pathway (reversible isomerization of MM-CoA and succinyl-CoA [S-CoA]) (28–30). These precursor metabolic pathways, typically exploited for rational optimization of polyketide biosynthesis, have been uncovered and rewired for titer improvement (31, 32).

In the erythromycin biosynthetic gene (*ery*) cluster of *Sac. erythraea*, 21 genes are arranged in eight major transcriptional units; however, there is no CSR-encoding gene in the *ery* cluster (28). Discovery and characterization of multiple types of transcription factors (TFs) in *Sac. erythraea* have gradually elucidated unusual molecular mechanisms of regulation for erythromycin biosynthesis (33–39). Although these TFs have been shown to repress or activate the *ery* cluster either directly or indirectly, insights into the regulatory networks governing erythromycin biosynthesis represent merely a tip of the iceberg, particularly in ligand-mediated signaling regulation. Here, we identified a TetR-like TF directly repressing the *ery* cluster and uncovered a novel regulatory mode in which P-CoA and MM-CoA serve not only as the polyketide starter and extender units but also as the ligands of AcrT to coordinate erythromycin biosynthesis. Furthermore, this model may be common to other polyketide-producing actinomycetes, such as Streptomyces avermitilis and Streptomyces coelicolor, in which the corresponding starter and extender units for avermectin or actinorhodin biosynthesis also serve as the ligands of AcrT homologs.

## RESULTS

### A novel regulator is discovered with high-affinity binding to the *eryAI* promoter.

**FIG 1:**
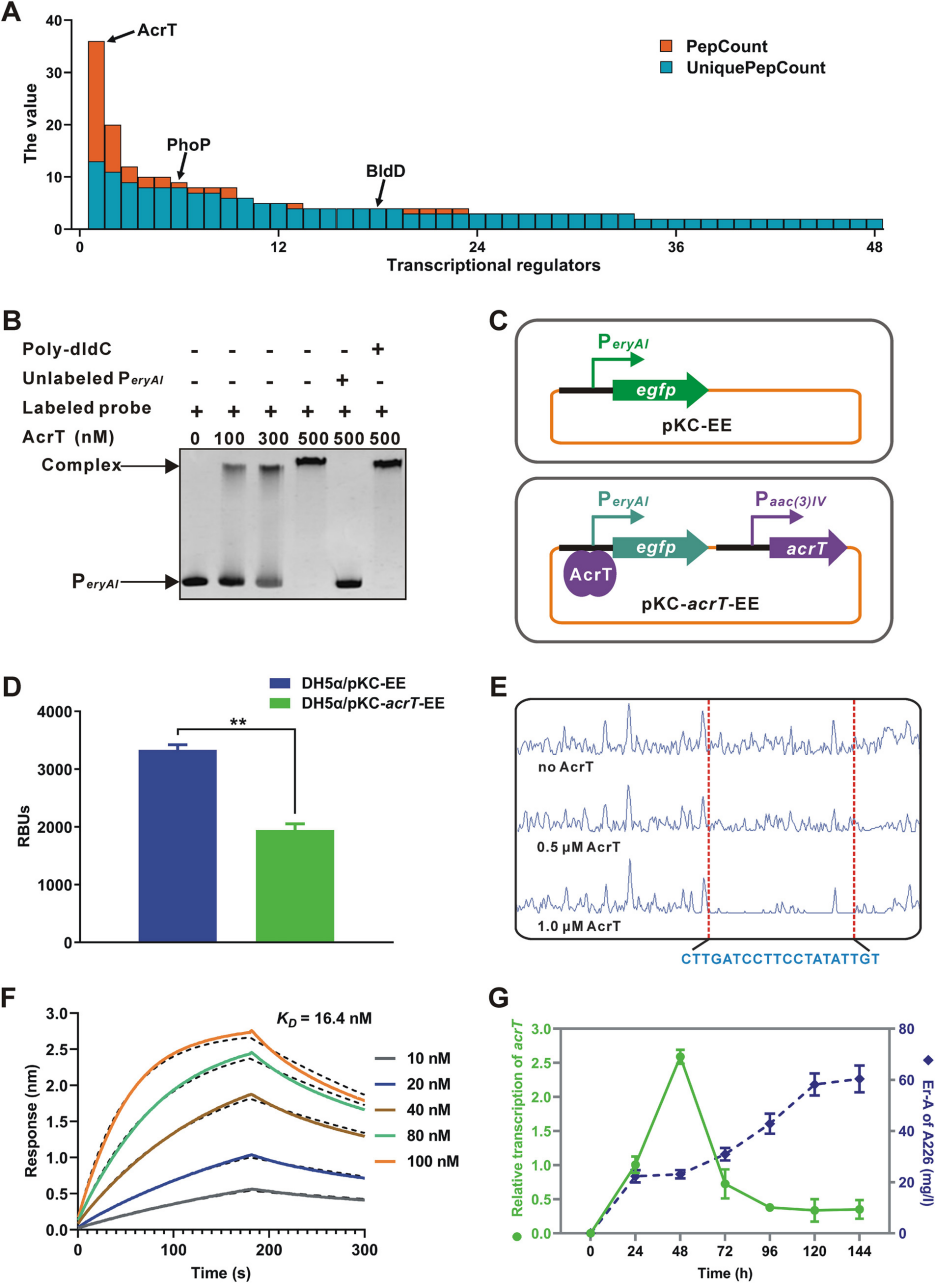
AcrT is a novel regulator interacting with P*_eryAI_*. (A) Screening of the potential P*_eryAI_*-interactive regulators by DACA. The PepCount and UniquePepCount values represent the total number of peptide fragments and the number of unique peptide fragments detected, respectively. Columns that are blue only indicate that the PepCount and UniquePepCount values were the same. Among those regulators, BldD and PhoP have been reported to bind to P*_eryAI_* in *Sac. erythraea* (33, 39). (B) EMSA of AcrT with P*_eryAI_*. Competing assays were performed using a 50-fold excess of unlabeled P*_eryAI_* or a 50-fold excess of nonspecific probe poly(dI-dC). (C) Illustration of the EGFP reporter system in E. coli DH5α. The system contained two plasmids, pKC-EE, expressing *egfp* under P*_eryAI_* without *acrT*, and pKC-*acrT*-EE, expressing *egfp* under P*_eryAI_* with *acrT* driven by the promoter of apramycin-resistance gene *aac*(3)*IV*. (D) Detection of relative bioluminescence units (RBUs) in E. coli DH5α/pKC-EE and DH5α/pKC-*acrT*-EE. (E) DNase I footprinting assay of the precise AcrT-binding site within P*_eryAI_*. (F) Affinity constant (*K_D_*) analysis by BLI. The chart shows the binding curves for the 50-bp probe within P*_eryAI_* against AcrT with different concentrations. (G) Time course of relative transcription of *acrT* and Er-A production in A226. Mean values of 3 measurements are shown with SDs. **, *P < *0.01.

The DNA affinity capture assay (DACA) is an *in vitro* method to directly capture DNA-binding TFs (40), which has been efficiently utilized to discover underlying regulatory pathways for the biosynthesis of antibiotics in actinomycetes (41, 42). Considering that the three *eryA* genes (*eryAI*, *eryAII*, and *eryAIII*) encoding PKSs for erythromycin biosynthesis are cotranscribed under the control of P*_eryAI_* (43), we used biotinylated P*_eryAI_* to isolate regulators interacting with the probe from total proteins of *Sac. erythraea* strain A226 (hereafter named A226). As determined by mass spectrometry (MS) analysis, 48 TFs mapped to >1 peptide fragments were identified as potential P*_eryAI_*-interactive regulators, in which AcrT (SACE_3980) possessed the highest number of detectable peptide fragments (Fig. 1A). Based on the genome annotation of *Sac. erythraea* (28), we found that AcrT is a TetR family transcriptional regulator and its homologs are widespread in polyketide-producing actinomycetes (see Fig. S1 in the supplemental material), suggesting that this type of TF has physiologically conserved regulatory roles.

10.1128/mBio.02298-21.1 FIG S1 Construction of neighbor-joining (NJ) distance tree of AcrT in *Sac. erythraea* and its homologs in polyketide-producing actinomycetes. The tree was constructed based on the amino acid sequences of AcrT and its homologs in polyketide-producing actinomycetes with MEGA (v6.06). Percentages represent the identities between AcrT and its homologs. The square, circle, and triangle stand for the bacteria studied in this work. Download FIG S1, PDF file, 0.3 MB. Copyright © 2021 Wu et al. 2021 Wu et al. https://creativecommons.org/licenses/by/4.0/ This content is distributed under the terms of the Creative Commons Attribution 4.0 International license.

Electrophoretic mobility shift assays (EMSAs) and the enhanced green fluorescent protein (EGFP) reporter system in Escherichia coli were used to investigate the regulatory pattern of AcrT acting on P*_eryAI_* (Fig. 1B to D). Results showed that AcrT specifically bound to P*_eryAI_ in vitro* (Fig. 1B; Fig. S2A), and bioluminescence was diminished with the expression of AcrT *in vivo* (Fig. 1D). Furthermore, a DNase I footprinting assay showed that a 20-nucleotide sequence, CTTGATCCTTCCTATATTGT (termed site A), was protected by AcrT (Fig. 1E). EMSAs with probes covering different sequences indicated that site A was indispensable for the binding of AcrT (Fig. S2B and C). Biolayer interferometry (BLI) assays confirmed that the interaction between the 50-bp fragment containing site A and AcrT had an affinity comparable to an equilibrium dissociation constant (*K_D_*) of 16.4 nM (Fig. 1F). Moreover, by monitoring *acrT* transcription and Er-A production in A226 during the fermentation successively with reverse transcription-quantitative PCR (RT-qPCR) and high-performance liquid chromatography (HPLC) analyses, we found that Er-A production was low and transcription of *acrT* appeared in the early stage, and as the yield of Er-A increased, *acrT* transcription peaked at 48 h and subsequently decreased to a very low level (Fig. 1G). Taken together, these results indicate that AcrT is a direct repressor of *eryAI* in the early stage of *Sac. erythraea* fermentation.

10.1128/mBio.02298-21.2 FIG S2 Determination of the precise site within P*_eryAI_* for AcrT to bind. (A) Identification of His-tagged AcrT by SDS-PAGE. (B) Illustration of the mutated probes. PU probe, 10 adjacent bases upstream of site A were mutated; PD probe, 10 adjacent bases downstream of site A were mutated; PM probe, site A was mutated. The mutated bases are marked with dashed boxes. (C) EMSA of AcrT binding to mutated probe PU, PD, or PM. Download FIG S2, PDF file, 0.3 MB. Copyright © 2021 Wu et al. 2021 Wu et al. https://creativecommons.org/licenses/by/4.0/ This content is distributed under the terms of the Creative Commons Attribution 4.0 International license.

### AcrT directly represses the transcription of *ery* cluster genes and its own gene.

**FIG 2:**
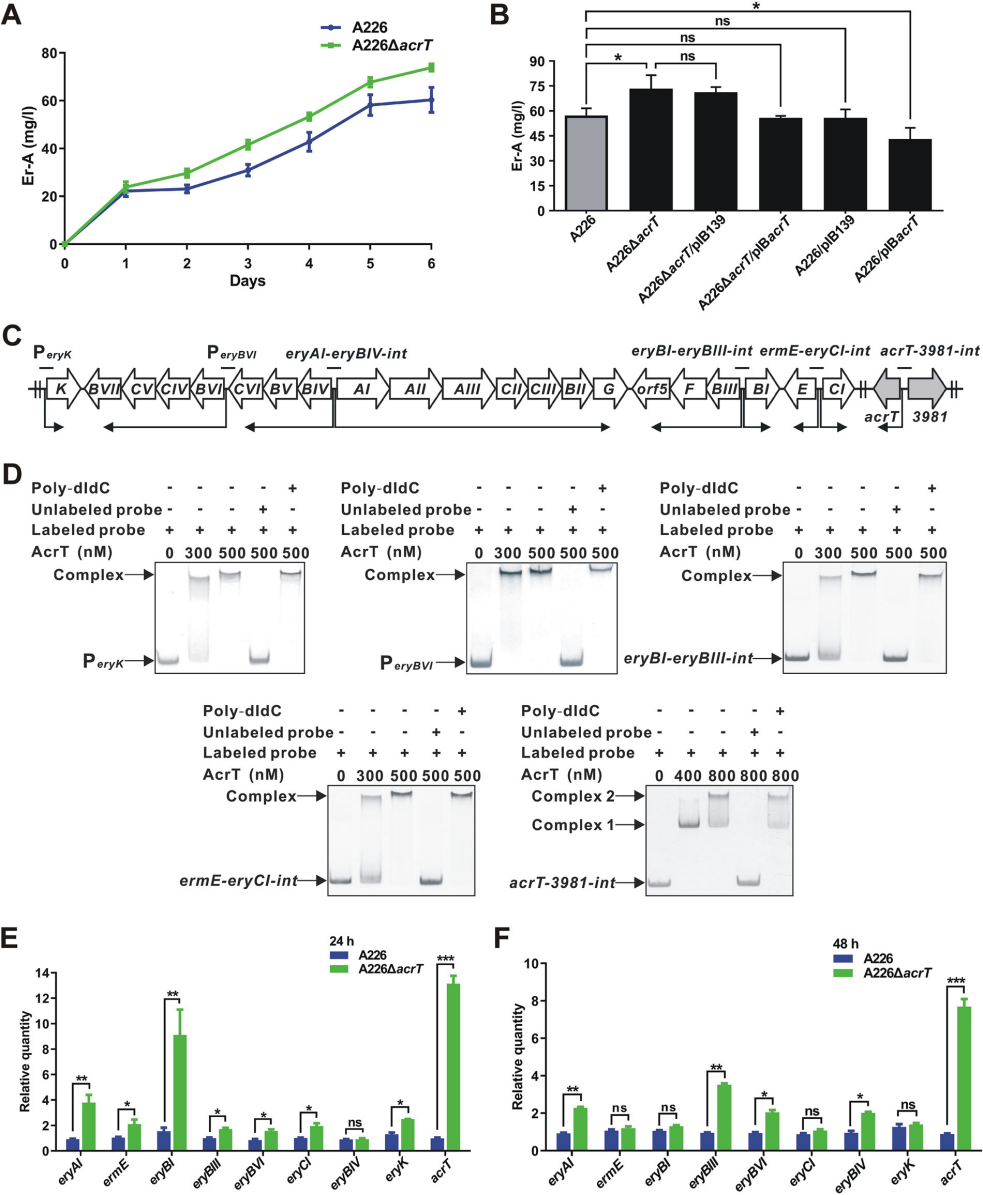
Erythromycin biosynthesis is directly repressed by AcrT. (A) Time course of Er-A production in A226 and A226Δ*acrT* by HPLC analyses. (B) Er-A production in A226 and its derivatives by HPLC analyses. (C) Genetic organization of *ery* cluster and *acrT* gene in *Sac. erythraea*. Black lines indicate the probes that contain promoters of the *ery* cluster genes. Bent arrows indicate the transcriptional unit and direction. (D) EMSAs with AcrT binding to the probes of P*_eryK_*, P*_eryBVI_*, *eryBI-BIII-int*, *ermE-eryCI-int*, and *acrT-3981-int*. Competing assays were performed using a 50-fold excess of unlabeled probes or a 50-fold excess of nonspecific probe poly(dI-dC). EMSA with AcrT binding to the probe of *eryAI-BIV-int* is shown in Fig. 1B with P*_eryAI_* instead. The two shifted bands (complex 1 and 2) were shown in EMSA with AcrT binding to *acrT-3981-int*, implying the existence of two AcrT-binding sites in *acrT-3981-int*. (E) RT-qPCR analyses of *ery* cluster genes and *acrT* in A226 and A226Δ*acrT* cultured for 24 h. (F) RT-qPCR analyses of *ery* cluster genes and *acrT* in A226 and A226Δ*acrT* cultured for 48 h. For these experiments, the mean values of 3 measurements are shown with SDs. *, *P < *0.05; **, *P < *0.01; ***, *P < *0.001; ns, not significant.

To investigate the function of AcrT, *acrT* was first disrupted with thiostrepton resistance gene (*tsr*) replacement in A226, which was confirmed by PCR (Fig. S3A and B). The difference in Er-A yield between A226 and A226Δ*acrT* appeared after 2 days of fermentation in R5 liquid medium, and A226Δ*acrT* showed a 28.3% increase in Er-A production compared to A226 on the 6th day (Fig. 2A and B). However, A226Δ*acrT* showed sporulation and growth rates similar to those of A226 (Fig. S3C and D). Complementation of *acrT* in A226Δ*acrT* pushed back Er-A production to its original level (Fig. 2B). Furthermore, when pIB*acrT* (see Table S1 in the supplemental material) was transformed into A226, the Er-A yield of A226/pIB*acrT* decreased by 20% compared to that of A226/pIB139 (Fig. 2B). Using the same method, we inactivated *acrT* in the industrial strain *Sac. erythraea* WB, and the Er-A yield of WBΔ*acrT* increased by 15.8% over that of WB (Fig. S3E and F). These results further suggest that AcrT functions as a repressor to control erythromycin biosynthesis in *Sac. erythraea*.

10.1128/mBio.02298-21.3 FIG S3 Inactivation of *acrT* in *Sac. erythraea*. (A) Schematic diagram of *acrT* deletion by homologous recombination with the linearized fragment in *Sac. erythraea* A226. (B) Identification of *acrT* deletion in *Sac. erythraea* A226. M, 5,000-bp DNA ladder; pUCTSRΔ*acrT*, the positive control, from which a 1,870-bp DNA fragment was amplified; A226, the negative control, from which a 756-bp DNA fragment was amplified; A226Δ*acrT*, the screened mutant, from which a 1,870-bp DNA fragment was amplified. (C) Aerial mycelium formation of A226 and its derivatives. All strains were grown on R3M solid medium at 30°C for 48 and 72 h. (D) Growth curves of A226 and A226Δ*acrT* in R5 liquid medium. The dry weights of mycelium (DWM) were measured. (E) Confirmation of *acrT* deletion in the industrial strain *Sac. erythraea* WB. M, 5,000-bp DNA ladder; pUCTSRΔ*acrT*, the positive control, from which a 1,870-bp DNA fragment was amplified; WB, the negative control, from which a 756-bp DNA fragment was amplified; WBΔ*acrT*, the screened mutant, from which a 1,870-bp DNA fragment was amplified. (F) Er-A production in WB and WBΔ*acrT*. Mean values of 3 measurements are shown with SDs. *, *P < *0.05. Download FIG S3, PDF file, 0.4 MB. Copyright © 2021 Wu et al. 2021 Wu et al. https://creativecommons.org/licenses/by/4.0/ This content is distributed under the terms of the Creative Commons Attribution 4.0 International license.

10.1128/mBio.02298-21.9 TABLE S1 Strains, plasmids, and primers used in this study. Download Table S1, PDF file, 0.4 MB. Copyright © 2021 Wu et al. 2021 Wu et al. https://creativecommons.org/licenses/by/4.0/ This content is distributed under the terms of the Creative Commons Attribution 4.0 International license.

Since AcrT had a high affinity for P*_eryAI_*, we wondered whether it interacted with other promoters within the *ery* cluster (Fig. 2C). EMSAs showed that AcrT also specifically binds to P*_eryK_*, P*_eryBVI_*, *eryBI-BIII-int*, and *ermE-eryCI-int* (Fig. 2D). Furthermore, we performed RT-qPCR experiments on eight genes within the *ery* cluster. The transcriptional levels of *eryAI*, *ermE*, *eryBI*, *eryBIII*, *eryBVI*, *eryCI*, *eryBIV*, and *eryK* in A226Δ*acrT* grown for 24 or 48 h exhibited overall increases compared with those in A226 (Fig. 2E and F). We also found that the *acrT* transcript markedly increased in A226Δ*acrT* cultured for both 24 and 48 h, respectively, in comparison to that in A226 (Fig. 2E and F). Moreover, EMSAs showed that AcrT specifically interacts with its own promoter (Fig. 2D). Therefore, our results verify that AcrT directly represses all genes in the *ery* cluster and itself.

### P-CoA and MM-CoA are ligands of AcrT for the regulation of *eryAI*.

Our recent investigations showed that ligands play pivotal roles in mediating the regulation of TFs for antibiotic biosynthesis in actinomycetes (37, 44, 45). Others reported that antibiotics or their biosynthetic intermediates as ligands can control their own biosynthesis by modulating the DNA-binding activity of TFs (8–14). Therefore, we tested whether Er-A and its biosynthetic intermediates could influence AcrT interacting with P*_eryAI_* and found that Er-A, Er-B, Er-C, and Er-D had no effect on AcrT binding to P*_eryAI_* (data not shown).

**FIG 3:**
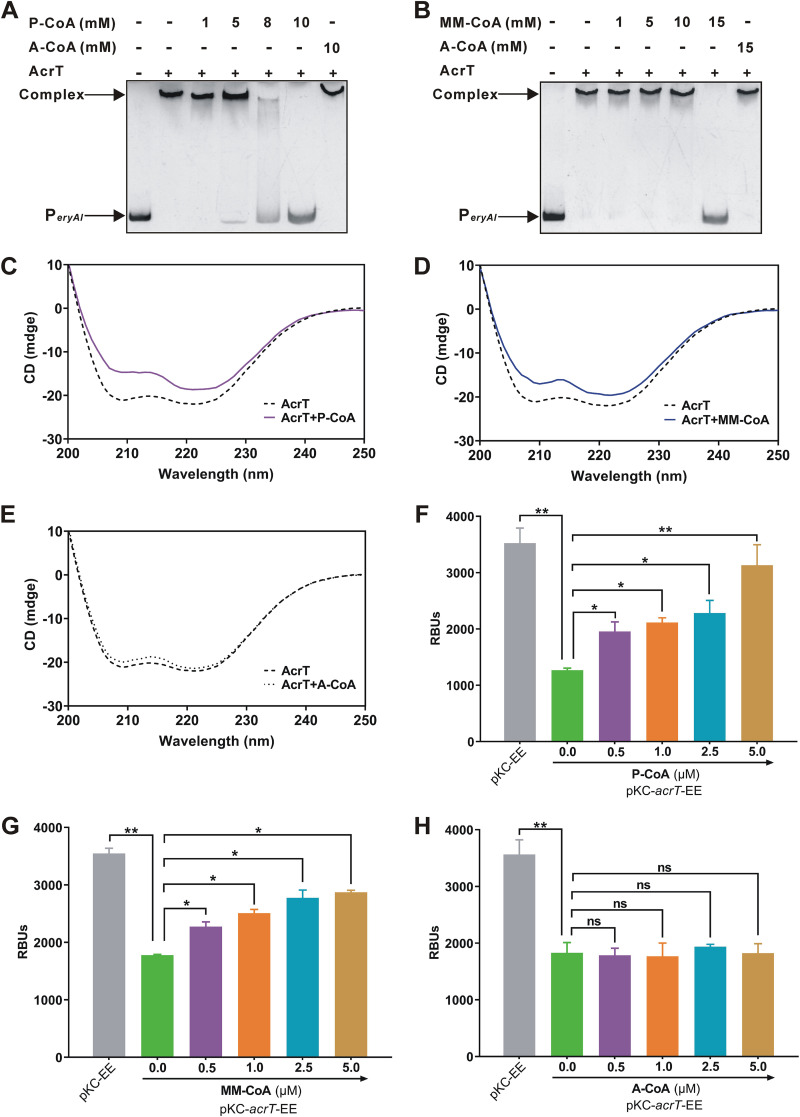
P-CoA and MM-CoA induce the dissociation of AcrT from P*_eryAI_*. (A) EMSAs of the effect of P-CoA on AcrT binding to P*_eryAI_*. A-CoA was used as the control. (B) EMSAs of the effect of MM-CoA on AcrT binding to P*_eryAI_*. A-CoA was used as the control. (C) CD spectra of AcrT in the absence and presence of P-CoA. The α-helix content of AcrT was characterized by two negative bands at 208 and 222 nm. The final concentration of P-CoA used here was 120 μM. (D) CD spectra of AcrT in the absence and presence of MM-CoA. The α-helix content of AcrT was characterized by two negative bands at 208 and 222 nm. The final concentration of MM-CoA used here was 120 μM. (E) CD spectra of AcrT in the absence and presence of A-CoA. The α-helix content of AcrT was characterized by two negative bands at 208 and 222 nm. A-CoA was used as the control. The final concentration of A-CoA used here was 120 μM. (F) EGFP reporter system to assay the interaction between P-CoA and AcrT. P-CoA was added to DH5α/pKC-*acrT*-EE. DH5α/pKC-EE was used as a control. (G) EGFP reporter system to assay the interaction between MM-CoA and AcrT. MM-CoA was added to DH5α/pKC-*acrT*-EE. DH5α/pKC-EE was used as a control. (H) EGFP reporter system to assay the interaction between A-CoA and AcrT. A-CoA was added to DH5α/pKC-*acrT*-EE. DH5α/pKC-EE was used as a control. Mean values of 3 measurements are shown with SDs. *, *P < *0.05; **, *P < *0.01; ns, not significant.

Since AcrT transcriptionally repressed *eryA* genes encoding PKSs to catalyze the condensation of P-CoA and MM-CoA, we explored effects of the two substrates on the binding ability of AcrT to P*_eryAI_*. It was demonstrated that P-CoA and MM-CoA could cause the dissociation of AcrT from P*_eryAI_*, and 10 mM P-CoA or 15 mM MM-CoA was sufficient for this, whereas A-CoA had no effect on the DNA-binding activity of AcrT (Fig. 3A and B). BLI assays also revealed that when 400 μM or 800 μM P-CoA was added, the affinity between AcrT and the 50-bp fragment containing site A was reduced from a *K_D_* of 16.4 nM without ligands to that of 81.1 nM or 194.0 nM, respectively (Fig. 1F; Fig. S4A and B). When MM-CoA was added at the same concentrations, the affinity dropped to a *K_D_* of 50.8 or 154.8 nM (Fig. S4C and D). Furthermore, the interactions between the two acyl-CoAs and AcrT were analyzed using circular-dichroism (CD) spectroscopy. Results showed that the α-helix content of AcrT markedly decreased after addition of P-CoA or MM-CoA, indicating that the two acyl-CoAs could interact with AcrT, while A-CoA did not obviously affect the signal intensity of AcrT (Fig. 3C to E). Subsequently, the EGFP reporter system in E. coli was used to investigate whether P-CoA and MM-CoA relieved the repression of AcrT on *eryAI* (Fig. 1C). When 0.5 to 5 μM P-CoA or MM-CoA was added to the system, bioluminescence was stimulated in a dose-dependent manner (Fig. 3F and G). The addition of A-CoA at the same concentrations made no differences in bioluminescence (Fig. 3H). Taken together, these findings corroborate that erythromycin biosynthetic starter and extender units, P-CoA and MM-CoA, play a novel role as effectors.

10.1128/mBio.02298-21.4 FIG S4 Affinity constant (*K_D_*) analysis by BLI. (A) Affinity with the addition of 400 μM P-CoA. (B) Affinity with the addition of 800 μM P-CoA. (C) Affinity with the addition of 400 μM MM-CoA. (D) Affinity with the addition of 800 μM MM-CoA. The chart shows the binding curves for the 50-bp probe within P*_eryAI_* against AcrT with different concentrations. Download FIG S4, PDF file, 0.3 MB. Copyright © 2021 Wu et al. 2021 Wu et al. https://creativecommons.org/licenses/by/4.0/ This content is distributed under the terms of the Creative Commons Attribution 4.0 International license.

### P-CoA and MM-CoA are increased by redistribution of A-CoA under AcrT inactivation.

**FIG 4:**
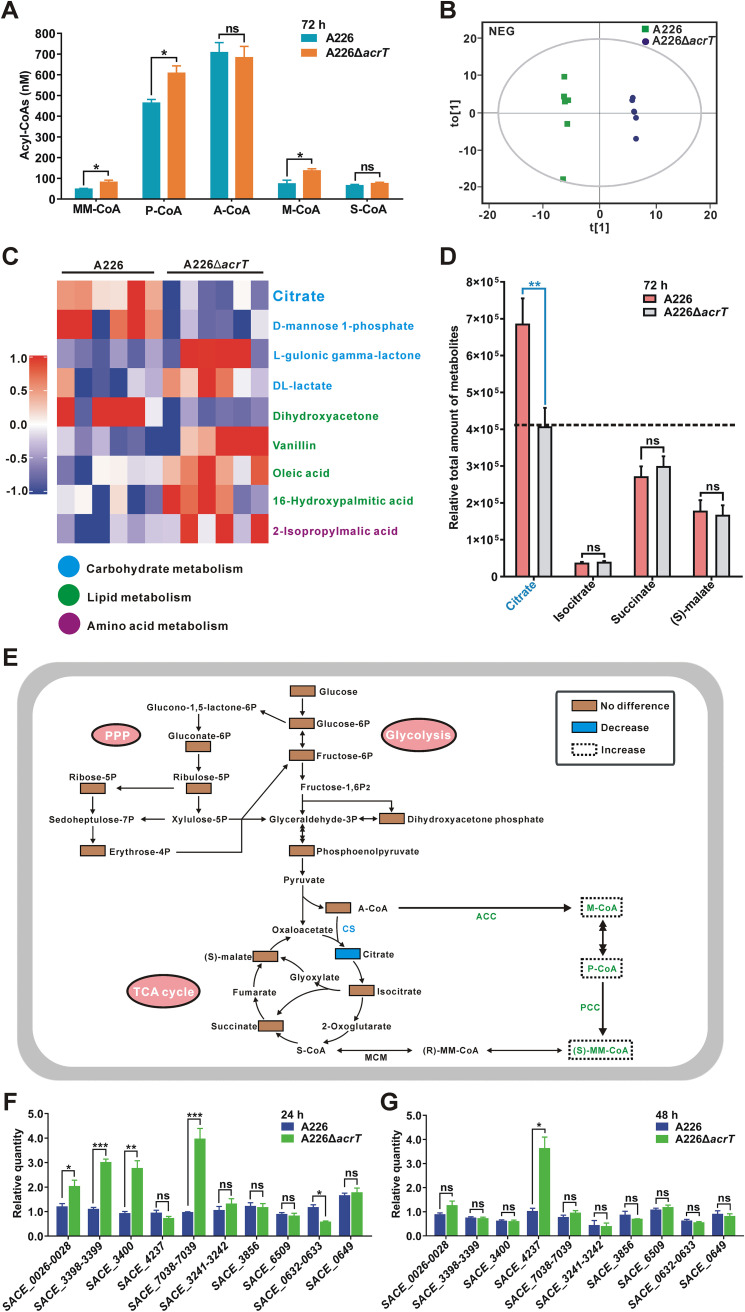
Enhancement of P-CoA and MM-CoA supplies via the redistribution of A-CoA metabolic flux. (A) Detection of intracellular concentrations of several acyl-CoAs in A226 and A226Δ*acrT* grown for 72 h. Mean values of 3 measurements are shown with SDs. (B) Orthogonal partial least-squares discrimination analysis (OPLS-DA) of the metabolic profiles in A226 and A226Δ*acrT* in negative ion mode. The analysis shows the ability to reliably predict and interpret the two sets of samples. (C) Hierarchical clustering analysis of metabolites of A226 and A226Δ*acrT* in negative ion mode. Some of metabolites which were significantly different between A226 and A226Δ*acrT* are summarized in the heat map. The colors of the squares represent the relative intensities of the metabolites in A226 and A226Δ*acrT*. The blue, green, and purple dots represent metabolites related to carbohydrate, lipid, and amino acid metabolism, respectively. (D) Relative total amounts of citrate, isocitrate, succinate, and (*S*)-malate in A226 and A226Δ*acrT*. Mean values of 6 measurements are shown with SDs. (E) Illustration of the intracellular supplies of P-CoA and MM-CoA from A-CoA after *acrT* deletion. The detectable metabolites from the glycolytic pathway (glycolysis), pentose phosphate pathway (PPP), and TCA cycle, through the metabolomic analysis of A226 and A226Δ*acrT* grown for 72 h, are marked with solid rectangular boxes, among which the citrate represented by the blue box changed and the others represented by brown boxes showed no differences. M-CoA, P-CoA, and MM-CoA marked with dotted boxes were enhanced. (F) Transcriptional levels of the ACC, PCC, and CS genes in A226 and A226Δ*acrT* cultured for 24 h. (G) Transcriptional levels of the ACC, PCC, and CS genes in A226 and A226Δ*acrT* cultured for 48 h. *SACE_0026-0028*, *SACE_3241-3242*, *SACE_3398-3399*, and *SACE_7038-7039* genes individually belong to the cotranscriptional unit (29). *SACE_0026-0028* and *SACE_3856* genes encode ACC enzymes; *SACE_3398-3399*, *SACE_7038-7039*, *SACE_3241-3242*, *SACE_3400*, *SACE_4237*, and *SACE_6509* genes encode ACC and/or PCC enzymes; *SACE_0632*, *SACE_0633*, and *SACE_0649* genes encode CS enzymes. Mean values of 3 measurements are shown with SDs. *, *P < *0.05; **, *P < *0.01; ***, *P < *0.001; ns, not significant.

Since P-CoA and MM-CoA were identified as the ligands of AcrT, we wondered if their metabolism was in turn controlled by AcrT. To this end, we compared the intracellular amounts of several acyl-CoAs between A226 and A226Δ*acrT*. As shown in Fig. 4A, the levels of M-CoA, P-CoA, and MM-CoA in A226Δ*acrT* were higher than those in A226, whereas A-CoA and S-CoA levels were not affected by *acrT* deletion. Hence, AcrT might control the biosynthesis of M-CoA, P-CoA, and MM-CoA via the ACC and PCC pathways. To verify this hypothesis, an untargeted multiple MS analysis was applied to profile intracellular metabolites within A226 and A226Δ*acrT* (Fig. 4B). In total, 348 metabolites from the major metabolic pathways were identified (Table S2A), among which 39 with variable influence on projection (VIP) values greater than 1.0 and *P* values less than 0.05 were considered to be significantly different (highlighted with blue in Table S2A), and some were involved in carbohydrate, lipid, and amino acid metabolism (Fig. 4C). Although the majority of detectable metabolites in the glycolytic pathway, the pentose phosphate pathway, and the tricarboxylic acid (TCA) cycle did not significantly change, the amount of citrate evidently decreased after the deletion of *acrT* (Fig. 4C to E). This implied that the enhancement of intracellular M-CoA, P-CoA, and MM-CoA might be derived from A-CoA, which previously flowed to citrate. Simultaneously, we noticed that the intracellular level of citrate was much higher than those of isocitrate, succinate, and (*S*)-malate in both strains (Fig. 4D), suggesting that citrate was sufficient to maintain the normal metabolism of the TCA cycle, even if some A-CoA turned to M-CoA, P-CoA, and MM-CoA instead of citrate.

10.1128/mBio.02298-21.10 TABLE S2 (A) Profiles of detectable metabolites in A226 and A226Δ*acrT* by metabolomic analysis. (B) Putative target genes of AcrT in *Sac. erythraea*. (C) Sources of materials or services in this study. Download Table S2, XLSX file, 0.06 MB. Copyright © 2021 Wu et al. 2021 Wu et al. https://creativecommons.org/licenses/by/4.0/ This content is distributed under the terms of the Creative Commons Attribution 4.0 International license.

We further compared the transcriptional levels of eight sets of putative ACC and/or PCC genes (*SACE_0026-0028*, *SACE_3241-3242*, *SACE_3398-3399*, *SACE_3400*, *SACE_3856*, *SACE_4237*, *SACE_6509*, and *SACE_7038-7039*) and three citrate synthase (CS) genes (*SACE_0632*, *SACE_0633*, and *SACE_0649*) between A226 and A226Δ*acrT* (28, 29). The results showed that the transcripts of *SACE_0026-0028*, *SACE_3398-3399*, *SACE_3400*, and *SACE_7038-7039* increased by 1.7-, 2.7-, 3.1-, and 4.1-fold in A226Δ*acrT* in comparison to A226 after growing for 24 h. The *SACE_4237* transcript increased by 3.6-fold in A226Δ*acrT* compared to A226 after growing for 48 h (Fig. 4F and G), whereas the transcripts of *SACE_0632-0633* (cotranscriptional unit demonstrated in Fig. S5A and B) decreased by 47% in A226Δ*acrT* cultured for 24 h relative to that in A226 (Fig. 4F). Based on these findings, we conclude that AcrT can manage the distribution of A-CoA metabolic flux via the differential modulation of ACC, PCC, and CS enzymes.

10.1128/mBio.02298-21.5 FIG S5 Interactions of AcrT with the promoters of ACC/PCC and CS genes. (A) PCR primer design of *SACE_0632-0633* (CS genes) for identifying the transcriptional unit. A solid line indicates the DNA fragment across *SACE_0632* and *SACE_0633* in A226. The negative number represents the overlapping region of these two genes. (B) Determination of transcriptional unit of *SACE_0632-0633*. Lane M, 5,000-bp DNA ladder; lane G, the PCR products using genomic DNA of A226 as the template; lane C, the PCR products using the cDNA library of A226 as the template. (C) PCR primer design of *SACE_0018-0026* genes for identifying the transcriptional unit. Solid lines indicate DNA fragments across the adjacent genes in A226. A negative number represents an overlapping region of two adjacent genes, and a positive number represents an intergenic region of two adjacent genes. (D) Determination of cotranscription of *SACE_0018-0026* genes. Here we determined that the real promoter of *SACE_0026-0028* genes was located upstream of *SACE_0018*, not upstream of *SACE_0026*. Lane M, 5,000-bp DNA ladder; lane G, the PCR products using genomic DNA of A226 as the template; lane C, the PCR products using cDNA library of A226 as the template. (E) EMSA with AcrT binding to P*_0018-0028_* (*SACE_0026-0028*, ACC genes). (F) EMSA with AcrT binding to P*_3400_* (*SACE_3400*, ACC or PCC gene). (G) EMSA with AcrT binding to P*_7038-7039_* (*SACE_7038-7039*, ACC and/or PCC genes). (H) EMSA with AcrT binding to P*_0632-0633_* (*SACE_0632-0633*, CS genes). (I) EMSA with AcrT binding to P*_3398-3399_* (*SACE_3398-3399*, ACC and PCC genes). (J) EMSA with AcrT binding to P*_4237_* (*SACE_4237*, ACC or PCC gene). (K) EMSA with AcrT binding to P*_3241-3242_* (*SACE_3241-3242*, ACC and/or PCC genes). (L) EMSA with AcrT binding to P*_0649_* (*SACE_0649*, CS gene). P*_3241-3242_* and P*_0649_* were used as the negative controls. Competing assays were performed using a 50-fold excess of unlabeled probes or a 50-fold excess of nonspecific probe poly(dI-dC). Download FIG S5, PDF file, 0.3 MB. Copyright © 2021 Wu et al. 2021 Wu et al. https://creativecommons.org/licenses/by/4.0/ This content is distributed under the terms of the Creative Commons Attribution 4.0 International license.

### P-CoA and MM-CoA can coordinate their own supplies for erythromycin biosynthesis.

**FIG 5:**
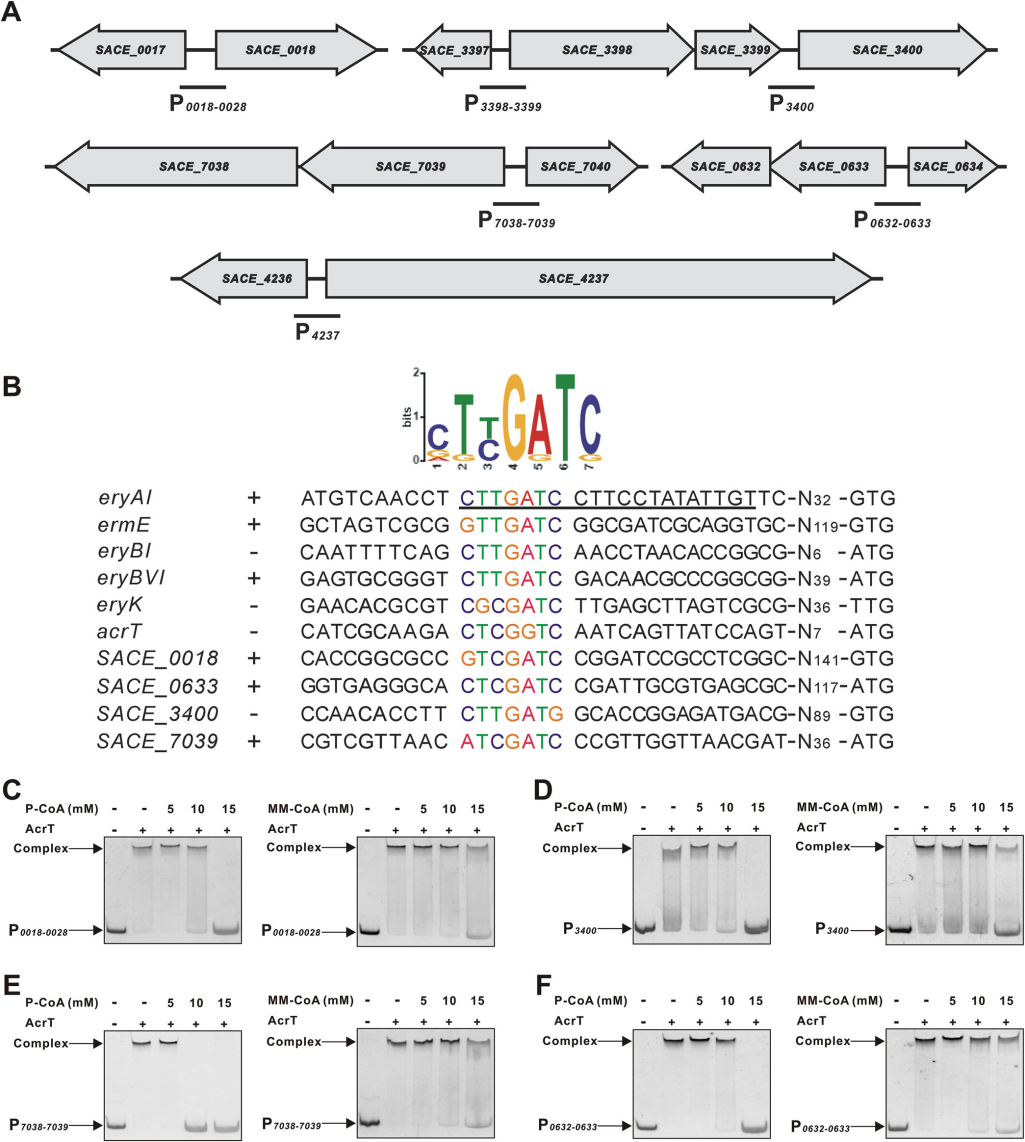
P-CoA and MM-CoA induce the dissociation of AcrT from promoters of the ACC, PCC, and CS genes. (A) Diagram of target probes for EMSAs. Black lines, probes containing the promoters of the target genes. (B) Analysis of the conserved AcrT-binding motif within the promoter regions of *eryAI*, *ermE*, *eryBI*, *eryBVI*, *eryK*, *SACE_0018-0028*, *SACE_0632-0633*, *SACE_3400*, and *SACE_7038-7039*. The standard code of the WebLogo server is shown at the top using online MEME software. The precise AcrT-binding site in P*_eryAI_* is underlined. (C) Effects of P-CoA and MM-CoA on AcrT binding to P*_0018-0028_*. (D) Effects of P-CoA and MM-CoA on AcrT binding to P*_3400_*. (E) Effects of P-CoA and MM-CoA on AcrT binding to P*_7038-7039_*. (F) Effects of P-CoA and MM-CoA on AcrT binding to P*_0632-0633_*.

To explore the regulatory pattern of AcrT with respect to these ACC, PCC, and CS genes (Fig. 5A; Fig. S5C and D), EMSAs were carried out. Results showed that AcrT specifically bound to P*_0018-0028_*, P*_3400_*, P*_7038-7039_*, and P*_0632-0633_* (Fig. S5E to H) but not to P*_3398-3399_* and P*_4237_*, as well as the negative controls P*_3241-3242_* and P*_0649_* (Fig. S5I to L). Using the motif-finding program MEME (http://meme-suite.org/) with the upstream sequences of those genes, a conserved AcrT-binding motif (nttGaTc, n: C, G, A; t: T, G/C; a: A, G; c: C, G) similar to site A was identified (Fig. 5B).

Since P-CoA and MM-CoA could dissociate AcrT from P*_eryAI_*, we wondered whether the two acyl-CoAs also affected the interaction between AcrT and P*_0018-0028_*, P*_3400_*, P*_7038-7039_*, or P*_0632-0633_*. EMSA results showed that P-CoA could efficiently pull AcrT down from the four probes, and MM-CoA had a bit weaker effect (Fig. 5C to F), while A-CoA had no any effect; however, P-CoA and MM-CoA did not influence AcrT binding to its own promoter (data not shown). Based on these findings, it is proved that P-CoA and MM-CoA, as ligands, can synthetically coordinate erythromycin biosynthesis by multiple approaches.

### Polyketide starter and extender units prevalently act as regulatory ligands.

Considering that the homologs of AcrT are widespread in the polyketide-producing actinomycetes (Fig. S1), we wanted to know whether the regulatory mechanism is universal.

**FIG 6:**
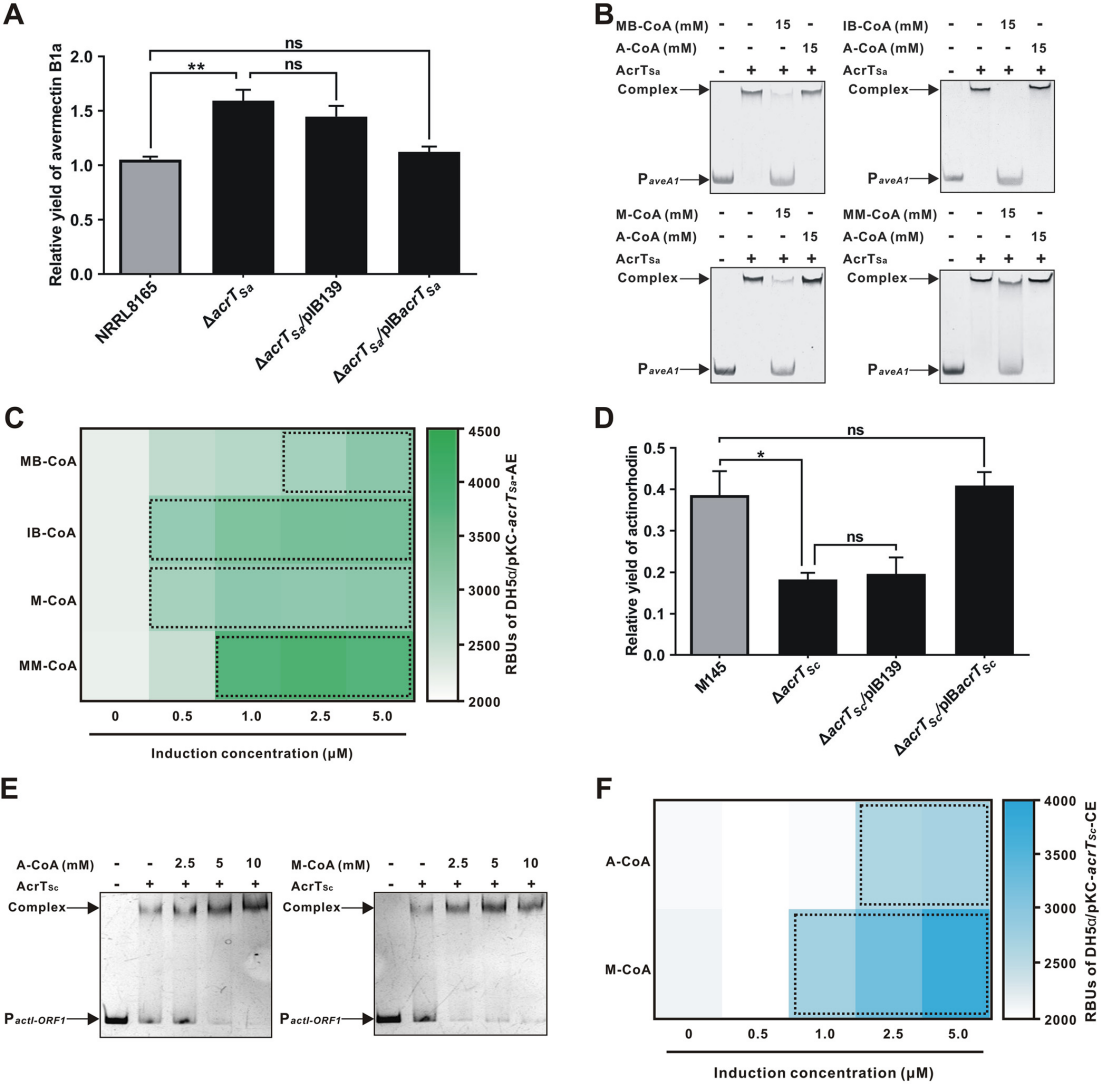
Polyketide starter and extender units mediate the regulation of avermectin and actinorhodin biosynthesis. (A) Avermectin B1a production in *S. avermitilis* NRRL8165 and its derivatives by HPLC analyses. The ratios of the production of NRRL8165 and its derivatives are shown. (B) Effects of the acyl-CoAs on AcrT_Sa_ binding to P*_aveA1_*. (C) RBUs with various concentrations of the four acyl-CoAs in the reporter system. pKC-*acrT_Sa_*-AE expresses *egfp* under P*_aveA1_* with *acrT_Sa_* driven by P*_aac(3)IV_*. The dotted boxes represent the RBUs with significant differences after the addition of the four acyl-CoAs. (D) Actinorhodin production in S. coelicolor M145 and its derivatives. The relative yields were quantified by spectrophotometry at 640 nm. (E) The effects of A-CoA and M-CoA on AcrT_Sc_ binding to P*_actI-ORF1_*. (F) RBUs with various concentrations of A-CoA or M-CoA in the reporter system. pKC-*acrT_Sc_*-CE expresses *egfp* under P*_actI-ORF1_* with *acrT_Sc_* driven by P*_aac(3)IV_*. The dotted boxes represent the RBUs with significant differences after the addition of the two acyl-CoAs. Mean values of 3 measurements are shown with SDs. *, *P < *0.05; **, *P < *0.01; ns, not significant.

Avermectin is a typical type I polyketide that is constructed using the starter unit methylbutyryl-CoA (MB-CoA) or isobutyryl-CoA (IB-CoA) and extender units M-CoA and MM-CoA in *S. avermitilis* (46). A TetR family regulator, SAV4017, here named AcrT_Sa_, shared 61% amino acid identity with AcrT (Fig. S1). Genetic experiments with disruption and complementation of *acrT_Sa_* demonstrated that AcrT_Sa_ negatively affected the production of avermectin B1a (Fig. 6A). Using EMSA, RT-qPCR, and EGFP reporter system experiments, we found that AcrT_Sa_ directly suppressed the transcription of *aveA1*, which encodes a PKS for avermectin biosynthesis (Fig. S6A to E), and that the four acyl-CoA precursors could mediate the dissociation of AcrT_Sa_ from the promoter of *aveA1* (P*_aveA1_*), whereas A-CoA had no effect on the DNA-binding activity of AcrT_Sa_ (Fig. 6B). With the individual addition of those four acyl-CoAs (0.5 to 5 μM), bioluminescence was stimulated for all in a dose-dependent manner, whereas the addition of A-CoA did not affect the intensity of bioluminescence (Fig. 6C; Fig. S6F to J). Therefore, these results indicate that MB-CoA, IB-CoA, M-CoA, and MM-CoA serve as ligands to coordinate avermectin biosynthesis.

10.1128/mBio.02298-21.6 FIG S6 The starter and extender units relieve the repression of AcrT_Sa_ on P*_aveA1_*. (A) Identification of His-tagged AcrT_Sa_ by SDS-PAGE. (B) EMSA of AcrT_Sa_ binding to P*_aveA1_*. Competing assays were performed using a 50-fold excess of unlabeled P*_aveA1_* or a 50-fold excess of nonspecific probe poly(dI-dC). (C) RT-qPCR analyses of *aveA1* in *S. avermitilis* NRRL8165 and the Δ*acrT_Sa_* strain cultured for 24 and 72 h. (D) Illustration of the EGFP reporter system. The system used two plasmids, pKC-AE expressing *egfp* under P*_aveA1_* without *acrT_Sa_* and pKC-*acrT_Sa_*-AE expressing *egfp* under P*_aveA1_* with *acrT_Sa_* driven by P*_aac(3)IV_*. (E) Detection of RBUs of the EGFP reporter system in E. coli DH5α. (F) Detection of RBUs in E. coli DH5α/pKC-*acrT_Sa_*-AE with MB-CoA. (G) Detection of RBUs in E. coli DH5α/pKC-*acrT_Sa_*-AE with IB-CoA. (H) Detection of RBUs in E. coli DH5α/pKC-*acrT_Sa_*-AE with M-CoA. (I) Detection of RBUs in E. coli DH5α/pKC-*acrT_Sa_*-AE with MM-CoA. (J) Detection of RBUs in E. coli DH5α/pKC-*acrT_Sa_*-AE with A-CoA (the control). Mean values of 3 measurements are shown with SDs. *, *P < *0.05; **, *P < *0.01; ***, *P < *0.001; ns, not significant. Download FIG S6, PDF file, 0.5 MB. Copyright © 2021 Wu et al. 2021 Wu et al. https://creativecommons.org/licenses/by/4.0/ This content is distributed under the terms of the Creative Commons Attribution 4.0 International license.

Moreover, we explored the ligand-mediated regulatory mode in the biosynthesis of actinorhodin, a type II polyketide in S. coelicolor. The TetR family regulator SCO4194, here named AcrT_Sc_, likewise had high amino acid identity (58%) with AcrT (Fig. S1). Correspondingly, AcrT_Sc_ was found to specifically bind to the promoter of *actI-ORF1* (P*_actI-ORF1_*), which encodes a PKS for actinorhodin biosynthesis (Fig. S7A and B). Distinct only from AcrT and AcrT_Sa_, AcrT_Sc_ acted as an activator to stimulate the transcription of the *actI-ORF1* gene (Fig. S7C to E) and displayed a positive correlation with actinorhodin production (Fig. 6D). As A-CoA and M-CoA are the starter and extender units in actinorhodin biosynthesis (47), we further explored whether they also influenced the binding activity of AcrT_Sc_ to P*_actI-ORF1_*. Results from EMSAs showed that A-CoA or M-CoA could promote AcrT_Sc_ to interact with P*_actI-ORF1_* (Fig. 6E). Bioluminescence was stimulated in a dose-dependent manner after the addition of 0.5 to 5 μM A-CoA or M-CoA to the EGFP reporter system (Fig. 6F; Fig. S7F and G). These results indicate that A-CoA and M-CoA also act as ligands to modulate the biosynthesis of actinorhodin.

10.1128/mBio.02298-21.7 FIG S7 The starter and extender units promote the activation of AcrT_Sc_ on P*_actI-ORF1_*. (A) Identification of His-tagged AcrT_Sc_ by SDS-PAGE. (B) EMSA of AcrT_Sc_ binding to P*_actI-ORF1_*. Competing assays were performed using a 50-fold excess of unlabeled P*_actI-ORF1_* or a 50-fold excess of nonspecific probe poly(dI-dC). (C) RT-qPCR analyses of *actI-ORF1* in S. coelicolor M145 and the Δ*acrT_Sc_* strain cultured for 24 and 48 h. (D) Illustration of the EGFP reporter system. The system used two plasmids, pKC-CE expressing *egfp* under P*_actI-ORF1_* without *acrT_Sc_* and pKC-*acrT_Sc_*-CE expressing *egfp* under P*_actI-ORF1_* with *acrT_Sc_* driven by P*_aac(3)IV_*. (E) Detection of RBUs of the EGFP reporter system in E. coli DH5α. (F) Detection of RBUs in E. coli DH5α/pKC-*acrT_Sc_*-CE with A-CoA. (G) Detection of RBUs in E. coli DH5α/pKC-*acrT_Sc_*-CE with M-CoA. Mean values of 3 measurements are shown with SDs. *, *P < *0.05; **, *P < *0.01; ***, *P < *0.001; ns, not significant. Download FIG S7, PDF file, 0.4 MB. Copyright © 2021 Wu et al. 2021 Wu et al. https://creativecommons.org/licenses/by/4.0/ This content is distributed under the terms of the Creative Commons Attribution 4.0 International license.

Therefore, our findings reveal that the starter and extender units function as ligands to allosterically modulate the DNA-binding activities of AcrT-like TFs, regardless of activators or repressors, ultimately promoting the biosynthesis of polyketides in actinomycetes.

## DISCUSSION

Acyl-CoAs are involved in more than 100 cellular reactions in various biological processes of microorganisms, including glycolysis, TCA cycle, metabolism of amino acids and fatty acids, and biosynthesis of secondary metabolites (48). It is well documented that certain acyl-CoAs, as original building blocks, can be condensed to generate diverse polyketides in actinomycetes (21). A recent report has shown that some acyl-CoAs are also functional as major donors in the acylation of biosynthetic enzymes to modulate the synthesis of natural products (26). Our present work unprecedentedly found that the starter unit P-CoA and extender unit MM-CoA act as the ligands of AcrT to construct the P-/MM-CoA**–**AcrT**–**PKS circuit coordinating the synthesis of erythromycin in *Sac. erythraea*. AcrT could control the supply of the two acyl-CoAs via distribution of A-CoA metabolic flux, which in turn was modulated by the two acyl-CoAs as ligands. Based on these data, as well as those from *S. avermitilis* and S. coelicolor, we conclude that polyketide starter and extender units universally play an alternative role as ligands to coordinate antibiotic biosynthesis in actinomycetes.

In recent years, several types of TFs have been investigated in *Sac. erythraea*; however, elucidation of the regulatory network governing erythromycin biosynthesis remains limited. DACA is a very effective strategy to capture and identify potential TFs (41, 42). Using this method, we identified at least 48 potential P*_eryAI_*-interactive TFs, among which BldD and PhoP have been reported to bind to P*_eryAI_* in *Sac. erythraea* (33, 39) (Fig. 1). Unfortunately, SACE_7301 and SACE_3446 (35, 36), other previously published TFs that directly interact with P*_eryAI_*, were not detected among these TFs. Furthermore, we compared the affinities of AcrT and these four TFs toward P*_eryAI_* and found that AcrT exhibited an affinity with a *K_D_* of 11 nM under our experimental conditions, which was approximately 15-, 20-, 22-, and 33-fold higher than values for SACE_3446 (165 nM), PhoP (219 nM), SACE_7301 (245 nM), and BldD (364 nM), respectively. Therefore, it seems necessary to further optimize the conditions of this strategy for a promoter to capture its every potential TF.

Our recent investigations have shown that there exist complex regulatory mechanisms in the biosynthesis of erythromycin (34–36, 38), and the ligands of TFs play nonnegligible roles in antibiotic biosynthesis (37, 44, 45). Antibiotics or their intermediates were previously found to function as ligands of TFs for feedback or feed-forward modulation in actinomycetes (3). Herein, for the first time, we verified that the direct precursors, P-CoA and MM-CoA, can coordinate the biosynthesis of erythromycin as ligands. An *in vivo* reporter system showed that 0.5 μM P-CoA or MM-CoA, which is approximately the physiological concentration (Fig. 4), can effectively relieve AcrT repressive effect on P*_eryAI_* (Fig. 3), suggesting that these two acyl-CoAs probably serve as signal molecules to promote erythromycin biosynthesis in *Sac. erythraea*. Very recently, methylcrotonyl-CoA, P-CoA, and A-CoA were found to be ligands of AccR in *S. avermitilis*, but they were not the starter and extender units for avermectin biosynthesis (49).

Moreover, our metabolomic analysis suggested that AcrT acts as a key coordinator to distribute the metabolic flux of A-CoA through the ACC and CS pathways. When *acrT* was deleted, A-CoA was more converted to M-CoA via the ACC path than via the CS path despite that it remained at relatively constant levels (Fig. 4). Although the level of citrate decreased in response to *acrT* deletion, it was still maintained at a high enough level to generate sufficient isocitrate, succinate, and (*S*)-malate as measured within the TCA cycle (Fig. 4). In *Sac. erythraea*, there are eight sets of putative ACC enzymes that may transform A-CoA to M-CoA and two CS enzymes that may transform A-CoA to citrate. RT-qPCR and EMSA analyses showed that AcrT directly represses three sets of ACC genes and stimulates one CS operon (Fig. 4; Fig. S5), inferring that there may be additional pathways to control the other ACC or CS genes. Therefore, AcrT might be one center to regulate A-CoA metabolic flux by the opposite modulation of the ACC and CS pathways, reducing P-CoA and MM-CoA and increasing citrate for cell growth at the early stage. Meanwhile, with the accumulation of P-CoA and MM-CoA, they in turn served as the ligands of AcrT to modulate more A-CoA metabolic flux from the CS path to the ACC path (Fig. 5), indicating that the two acyl-CoAs also coordinate their own metabolism to supply more precursors for erythromycin biosynthesis.

**FIG 7:**
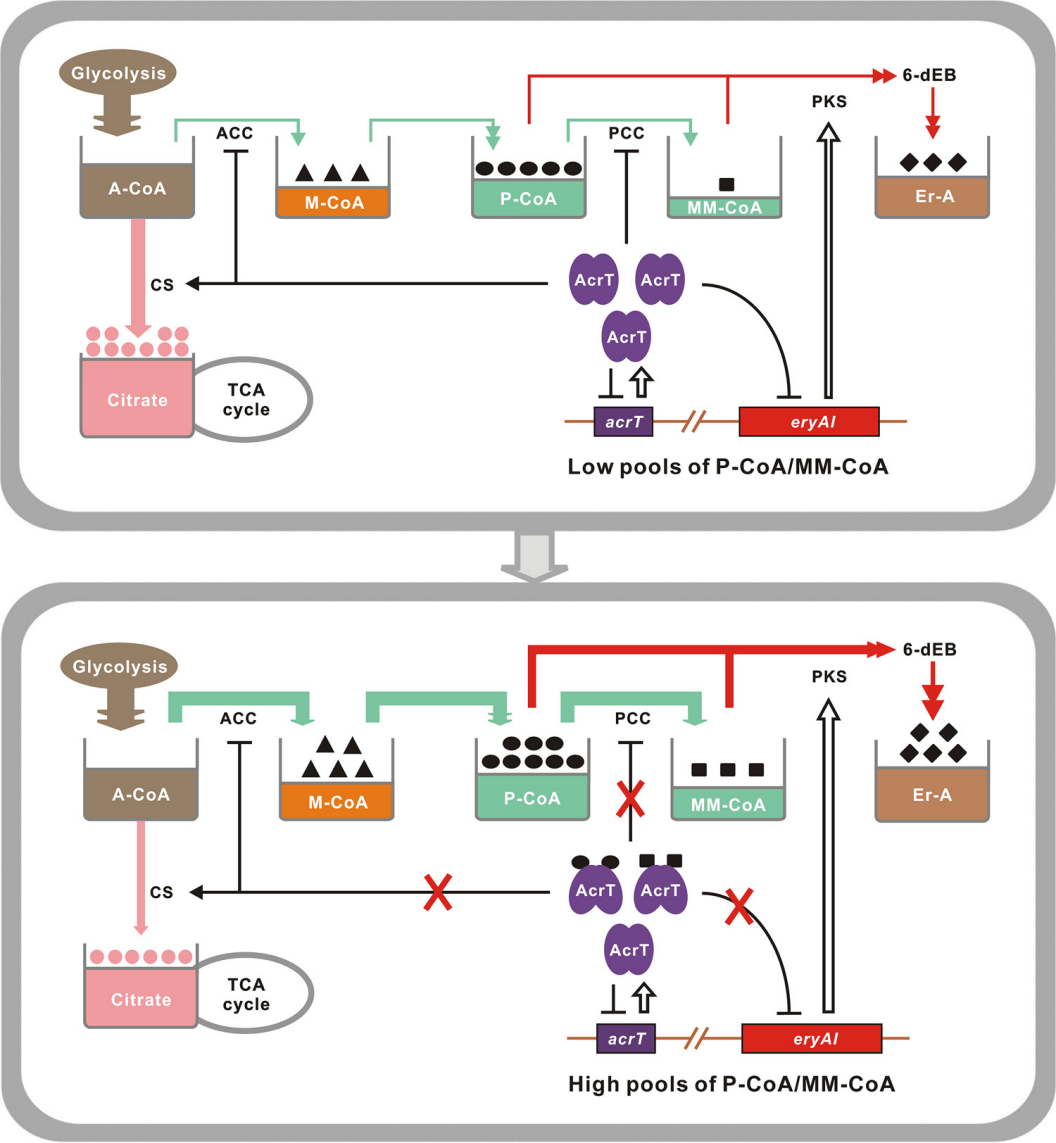
P-CoA and MM-CoA serve as the ligands of AcrT to coordinate erythromycin biosynthesis in *Sac. erythraea*. When the intracellular P-CoA and MM-CoA pools are initially at low levels, AcrT interacts with the promoters of *eryAI*, ACC, PCC, and CS genes to inhibit the production of erythromycin and stimulate the biosynthesis of citrate beneficial to cell growth. With bacterial growth, the two acyl-CoAs accumulate enough to dissociate AcrT from those target promoters, increasing their own metabolic pools and turning to produce more erythromycin. ACC, A-CoA carboxylase; PCC, P-CoA carboxylase; CS, citrate synthase; 6-dEB, 6-deoxyerythronolide B; Er-A, erythromycin A. Double arrows represent reactions of two steps or more. White-filled arrows represent translation. Black arrows represent transcriptional activation. Black blocked line represents transcriptional inhibition. Interruption is indicated by a red X.

Based on these findings, we propose a regulatory model for polyketide starter and extender units as ligands to coordinate erythromycin production (Fig. 7).

Furthermore, according to the precisely identified site of AcrT binding, we employed the PREDetector software to predict potential AcrT-binding target genes across the *Sac. erythraea* genome. A total of 359 putative target genes (cutoff score, ≥ 8) were identified (Fig. S8 and Table S2B), among which 91 were functionally unassigned and the remaining 268 were divided into 19 categories involved in major metabolic pathways, such as transport and metabolism of carbohydrates and amino acids, as well as metabolism of lipids. This implies that AcrT plays a global regulatory role.

10.1128/mBio.02298-21.8 FIG S8 Classification of putative target genes of AcrT based on the COG database of *Sac. erythraea*. Download FIG S8, PDF file, 0.3 MB. Copyright © 2021 Wu et al. 2021 Wu et al. https://creativecommons.org/licenses/by/4.0/ This content is distributed under the terms of the Creative Commons Attribution 4.0 International license.

To explore the universality of polyketide starter and extender units acting as ligands in polyketide-producing actinomycetes, we first chose *S. avermitilis*, which produces the type I polyketide avermectin. As expected, the starter unit (MB-CoA or IB-CoA) and extender units (M-CoA and MM-CoA) were the ligands of AcrT_Sa_, a homolog of AcrT, and suppressed the biosynthesis of avermectin (Fig. 6). Subsequently, we assessed the type II polyketide producer S. coelicolor and confirmed that the starter unit A-CoA and extender unit M-CoA were also the ligands of AcrT_Sc_, which was also homologous to AcrT, except that AcrT_Sc_ exhibited activation of actinorhodin biosynthesis (Fig. 6). Interestingly, in the biosynthesis of type I polyketides erythromycin and avermectin, AcrT and AcrT_Sa_ showed the same regulatory pattern, whereas AcrT_Sc_ exhibited an opposite effect on type II polyketide actinorhodin biosynthesis, indicating that AcrT homologs play complicated regulatory roles in transcriptional suppression or activation of antibiotic biosynthesis in actinomycetes. However, whether AcrT homologs suppress or activate the targets, the precursors as ligands are always beneficial to promote the biosynthesis of polyketides in actinomycetes.

All of our findings expand the knowledge that polyketide starter and extender units, beyond building blocks, play a vital role in coordinating the biosynthesis of antibiotics and enrich our understanding of the regulatory network in actinomycetes.

## MATERIALS AND METHODS

### Materials and culture conditions.

The bacterial strains, plasmids, and primers used in this study are listed in Table S1 in the supplemental material. The sources of enzymes, chemicals, reagents, primers, and DNA sequencing services are shown in Table S2C. E. coli strains were grown in Luria-Bertani (LB) medium (in g/liter: yeast extract, 5; tryptone, 10; NaCl, 10) at 37°C. E. coli DH5α was used for DNA cloning, E. coli BL21(DE3) for heterologous protein expression, and E. coli ET12567(pUZ8002) as the donor host for plasmid conjugation (50). *Sac. erythraea* A226, WB, and their derivatives were grown at 30°C on solid R3M medium (in g/liter: sucrose, 103; tryptone, 4; yeast extract, 4; Casamino Acids, 4; K_2_SO_4_, 0.25; and agar, 22; plus 20 ml 50% glucose, 20 ml 36.75% CaCl_2_·2H_2_O, 20 ml 50.75% MgCl_2_·6H_2_O, 12.5 ml 24.2% Tris-HCl [pH 7.0], 2.5 ml 4% NaOH, 370 μl 6.8% KH_2_PO_4_, and 200 μl trace element solution [g/liter: ZnCl_2_, 0.04; FeCl_3_·6H_2_O, 0.2; Na_2_B_4_O_7_·10H_2_O, 0.01; (NH_4_)_6_Mo_7_O_24_·4H_2_O, 0.1; MnCl_2_·4H_2_O, 0.1; and CuCl_2_·2H_2_O, 0.1]) for sporulation and protoplast manipulation, and liquid tryptic soy broth (TSB) medium (30 g/liter tryptone soy broth powder) was used for DNA extraction and protoplast preparation (37). *S. avermitilis* NRRL8165 and its derivatives were grown at 28°C on solid soy flour mannitol (SFM) (g/liter: soybean flour, 20; mannitol, 20; and agar, 20) and RM14 [g/liter: sucrose, 200; yeast extract, 5; Casamino Acids, 0.1; MgCl_2_·6H_2_O, 10.12; K_2_SO_4_, 0.25; and agar, 20; plus 10 ml 0.5% KH_2_PO_4_, 80 ml 3.68% CaCl_2_·2H_2_O, 15 ml 20% l-proline, 100 ml 5.73% 2-(4-morpholino) ethanesulfonic acid (MES), 5 ml 4% NaOH, and 2 ml trace element solution) media for sporulation and protoplast transformation or in liquid TSBY (g/liter: tryptone soy broth powder, 30; sucrose, 103; and yeast extract, 5) and YEME (g/liter: sucrose, 340; glucose, 10; yeast extract, 3; malt extract, 3; and tryptone, 5; plus 2 ml 23.75% MgCl_2_) media for DNA extraction and protoplast preparation, respectively (51). S. coelicolor M145 and its derivatives were grown at 30°C on solid SFM medium for sporulation and intergeneric conjugation or in liquid TSBY medium for DNA extraction (44).

### DACA.

Based on a previously described protocol (41), DACA was performed with minor revisions. The cells of A226 grown in R5 liquid medium [g/liter: sucrose, 103; glucose, 10; yeast extract, 5; Casamino Acids, 0.1; K_2_SO_4_, 0.25; MgCl_2_·6H_2_O, 10.12; and N-tris(hydroxymethyl) methyl-2-aminoethanesulfonic acid (TES), 5.73; plus 10 ml 0.5% KH_2_PO_4_, 4 ml 73.5% CaCl_2_·2H_2_O, 15 ml 20% l-proline, 7 ml 4% NaOH, and 2 ml trace element solution) for 48 and 72 h were harvested and resuspended in 100 ml binding buffer (10 mM Tris-HCl [pH 8.0], 5 mM MgCl_2_, 60 mM KCl, 10 mM dithiothreitol [DTT], 50 mM EDTA, and 10% glycerol) containing lysozyme solution at a final concentration of 2 mg/ml. The cells were then broken using an ultra-high-pressure crusher (JN-02C; JNBIO), followed by centrifugation (13,000 × *g*, 30 min, 4°C) to obtain the supernatant.

The biotinylated P*_eryAI_* probe or pUC probe (negative control) was amplified using the genome of A226 or pUC18 plasmid with biotin-labeled primers B-*eryAI*-F/R or B-pUC-F/R, respectively. Probes (20 μg) were individually added to 1 ml streptavidin-agarose (SA) (GE) and incubated in the binding buffer at room temperature for 30 min. The biotinylated pUC probe coupled with SA was first incubated with the total proteins extracted from A226 for 30 min at 30°C, followed by centrifugation (3,500 × *g*, 5 min, 4°C) to remove nonspecific binding proteins. The supernatant was then incubated with biotinylated P*_eryAI_*-SA supplemented with protease inhibitor (Roche) and sonicated salmon sperm DNA (Solarbio) for 60 min at 30°C. The bound proteins were eluted with elution buffer (binding buffer plus 1 M NaCl), isolated by sodium dodecyl sulfate-polyacrylamide gel electrophoresis (SDS-PAGE), and identified by a liquid chromatography-tandem mass spectrometry (LC-MS/MS) system consisting of the Easy-nLC 1000 system (Thermo Fisher Scientific) and Q Exactive mass spectrometer (Thermo Fisher Scientific) at Shanghai Applied Protein Technology Co., Ltd. The obtained data were searched against the UniProt database of *Sac. erythraea* NRRL2338.

### Heterologous expression and purification of proteins.

A DNA fragment encoding 224 amino acids of *acrT* was generated using the A226 genome as the template with the primers *acrT*-28a-F/R. The PCR product was digested with NdeI/HindIII restriction enzymes and cloned into pET28a to generate pET28a*acrT* with an N-terminal His tag. The plasmid was introduced into E. coli BL21(DE3) for the expression of AcrT. Likewise, AcrT_Sa_ and AcrT_Sc_ were expressed in E. coli BL21(DE3). The recombinant His-tagged proteins were purified on Ni^2+^-nitrilotriacetic acid (Ni^2+^-NTA) spin columns (Bio-Rad). The purified proteins were identified by SDS-PAGE and quantified using a bicinchoninic acid protein assay kit (Thermo Fisher Scientific).

### EMSAs.

EMSAs were carried out according to previously described methods (52). DNA probes were amplified by PCR using the primers listed in Table S1 and independently mixed with purified AcrT, AcrT_Sa_, and AcrT_Sc_ in the binding buffer to generate a 20-μl reaction mixture at 30°C for 20 min. After incubation, the reactants were separated on 6% native PAGE gels with 1× Tris-acetate-EDTA buffer as a running buffer at 60 mA for 40 to 50 min.

### EGFP reporter system in E. coli.

Based on a previously described protocol (45), the reporter system was constructed to test the interaction between AcrT and P*_eryAI_*. P*_eryAI_* and *egfp* were amplified using the primer pairs EE-F1/R1 and EE-F2/R2 and were cloned into the HindIII/BamHI sites of pKC1139 (53), creating pKC-EE. The *aac*(3)*IV* promoter and *acrT* were amplified with the primer pairs EE-F3/R3 and *acrT*-EE-F/R and inserted into the corresponding EcoRV/EcoRI sites of pKC-EE to generate pKC-*acrT*-EE. Likewise, the reporter systems for testing AcrT_Sa_ and AcrT_Sc_ functions were constructed as described previously herein.

These plasmids were introduced into E. coli DH5α. When concentrations of recombinant E. coli strains reached an optical density at 600 nm (OD_600_) of 0.8 to 1.0, the intensity of green fluorescence (excitation at 485 nm and emission at 510 nm; Molecular Devices) was measured and relative bioluminescence units (RBUs) were calculated by normalization to the growth rates (OD_600_). To estimate the interactions between AcrT, AcrT_Sa_, or AcrT_Sc_ and their corresponding ligands, acyl-CoAs at final concentrations of 0.5 to 5.0 μM were individually added into the reporter system.

### DNase I footprinting assay.

A nonradiochemical capillary electrophoresis method was used for DNase I footprinting (54). To determine the precise binding site of AcrT in *eryAI-eryBIV*-*int*, a 224-bp DNA fragment dually labeled with 5′-FAM and 5′-HEX was prepared using the primer pair FAM-*eryAI-BIV*-F/HEX-*eryAI-BIV*-R. The labeled DNA fragment (300 ng) and various concentrations of AcrT were incubated in a 50-μl total volume at 30°C for 30 min. DNase I (1 U/μg, Promega) digestion was carried out at 25°C for 60 s and stopped by adding DNase I Stop Solution (Promega) and heating at 65°C for 10 min. After purification, the samples were detected with a 3730XL DNA analyzer (Applied Biosystems), and data analyses were performed using the GeneMarker v2.2 software program.

### BLI analyses.

The binding affinity between the regulator (AcrT, PhoP, BldD, SACE_7301, or SACE_3446) and P*_eryAI_* were detected using the Octet K2 system with SA sensors (ForteBio) as previously described (55). The biotinylated P*_eryAI_* was obtained with the biotin-labeled primers BLI-*eryAI*-F1/R1 and immobilized on SA-coated biosensor tips. The reactions were conducted at 25°C in a buffer (1 mM Tris-HCl [pH 8.0], 5 mM MgCl_2_, 60 mM KCl, 10 mM DTT, 50 mM EDTA, and 10% glycerol), and the tips were immersed into wells containing purified proteins with appropriate concentration gradients. The data were set to an average model to determine the kinetic parameters *K*_on_ and *K*_off_. The binding affinities (*K_D_*) were then estimated as a ratio (*K*_off_/*K*_on_) of the rate constants. Based on the DNase I footprinting assay, a 50-bp biotinylated probe containing site A (the precise binding site of AcrT) was obtained by directly annealing the biotin-labeled primers BLI-*eryAI*-F2/R2, and the *K_D_* value was calculated according to the same procedure.

### Gene deletion, complementation, and overexpression.

Gene deletion, complementation, and overexpression in *Sac. erythraea* were performed as previously described (34). Two 1.5-kb DNA fragments flanking *acrT* were successively obtained using the primer pairs *acrT*-F1/R1 and *acrT*-F2/R2 with the A226 genome as the template. The amplified fragments were digested, individually, with XbaI/HindIII and EcoRI/KpnI restriction enzymes and ligated into the corresponding sites of pUCTSR to obtain pUCΔ*acrT*. Through the homologous recombination of linear fragments, a 394-bp fragment within *acrT* was replaced by *tsr* in A226. Using the primers *acrT*-F3/R3, the desired thiostrepton-resistant mutant, named A226Δ*acrT*, was confirmed by PCR. A 639-bp *acrT* fragment was amplified with the primers *acrT*-F4/R4 and cloned into the NdeI/XbaI sites of pIB139 (54) to generate pIB*acrT*. Then, the complementation strain A226Δ*acrT*/pIB*acrT* and the overexpression strain A226/pIB*acrT* were obtained by apramycin resistance screening, and the strains A226Δ*acrT*/pIB139 and A226/pIB139 were used as the controls. Similarly, *acrT* was disrupted in the industrial *Sac. erythraea* strain WB, generating WBΔ*acrT.*

*S. avermitilis* mutant construction was done in light of the procedure described previously herein with minor revisions. A 3.6-kb fragment containing *tsr* and the two homologous arms was ligated into the HindIII/EcoRI sites of pKC1139 (53). The obtained pKCΔ*acrT_Sa_* was introduced into *S. avermitilis* NRRL8165 and integrated into the chromosome by single crossover recombination. The strain could lose the plasmid at 37°C, generating the Δ*acrT_Sa_* mutant. The Δ*acrT_Sa_*/pIB*acrT_Sa_* complementation strain and Δ*acrT_Sa_*/pIB139 control strain were likewise obtained.

The construction of the S. coelicolor mutant was done by the intergeneric conjugation method (44). Similarly, pKCΔ*acrT_Sc_* was obtained as described previously herein and introduced into E. coli ET12567(pUZ8002). The strain was mixed with S. coelicolor M145 and cocultured on solid SFM medium, followed by coating with sodium naphthyridine and apramycin, generating a single crossover strain. The Δ*acrT_Sc_* mutant was subsequently obtained through temperature change as described for *S. avermitilis.* Based on intergeneric conjugation, the Δ*acrT_Sc_*/pIB*acrT_Sc_* complementation strain and Δ*acrT_Sc_*/pIB139 control strain were constructed.

### Antibiotic fermentation and measurement.

For flask fermentation of *Sac. erythraea* A226 and its derivatives, spores from R3M agar plates cultured for 3 days were inoculated into 50 ml TSB seed medium and shaken at 220 rpm at 30°C for 48 h. Five milliliters of culture was transferred into 50 ml of R5 liquid medium and grown for 6 days. *Sac. erythraea* strain WB and its derivative were cultivated in 50 ml of industrial fermentation medium [g/liter: cornstarch, 40; dextrin, 30; soybean flour, 30; soybean oil, 10; (NH_4_)_2_SO_4_, 2; CaCO_3_, 6). After 24 h of fermentation, *n*-propanol (1.0 ml) was added to the broth, which was further shaken for 5 days at 30°C. Using a previously described method (35), Er-A was extracted from the fermentation culture and quantified by HPLC analysis.

Flask fermentation of *S. avermitilis* NRRL8165 and its derivatives was carried out as previously described (51). Mycelia grown in SFM medium for 7 days were first inoculated into 50 ml of liquid seed medium (g/liter: cornstarch, 30; peanut powder, 10; soybean flour, 8; and yeast extract, 4; plus 3 ml 1% CoCl_2_) at 28°C for 2 days. Then, 2.5 ml culture was transferred into 50 ml of liquid fermentation medium [g/liter: cornstarch, 140; soybean flour, 28; amylase, 0.14; yeast extract, 10; and CaCO_3_, 0.8; plus 5 ml 5% (NH_4_)_2_SO_4_, 2 ml 1% CoCl_2_, 2.2 ml 1% Na_2_MoSO_4_, and 2.3 ml 1% MnSO_4_], and shaken with 220 rpm at 28°C for 10 days. Avermectin B1 was extracted using 9 volumes of methanol. The products were detected using an HPLC (Waters), which was equipped with an Extend-C_18_ column (5 μm, 4.6 by 150 mm; Shimadzu), and eluted with a mixture of 90% solution A (methanol) and 10% solution B (water). A program was performed with a flow rate of 1.0 ml/min and a UV detector at 246 nm.

Flask fermentation of S. coelicolor M145 and its derivatives was performed as previously described (44). Well-grown spores were first inoculated into 50 ml of TSBY medium at 30°C for 2 days. Five milliliters of culture was transferred into 50 ml of R5 liquid medium and further cultured for 7 days. To measure the actinorhodin yields of M145 and its derivatives, fermentation broths were treated with KOH solution at a final concentration of 1 M. After centrifugation (14,000 × *g*, 5 min, 4°C), the supernatant was quantified at a 640-nm wavelength. The level of actinorhodin was normalized to the biomass of mycelia.

### RNA preparation and RT-qPCR assays.

Using a TransZol kit (Transgen), total RNA was prepared from bacteria in liquid fermentation medium at different time points. The quality and quantity of RNA were examined using a microplate reader (BioTek) and confirmed by electrophoresis. RNA samples were treated by reverse transcription using a HiScript II Q RT supermix (Vazyme) to obtain cDNAs for RT-qPCR. The assays were performed on the QuantStudio 6 Flex system (Applied Biosystems), using a Maxima SYBR green/ROX qPCR master mix (Vazyme). The experiments were carried out with three technical replicates and three independent biological replicates. Endogenous *hrdB* was used as a control. The transcript levels of various genes were determined according to the manufacturer’s instructions.

### CD spectroscopy.

CD spectroscopic assays were recorded with a bandwidth of 2 nm at 25°C on a MOS-500 spectropolarimeter (Biologic) within the wavelength range of 200 to 250 nm. The protein was dissolved in 50 mM phosphate buffer solution (pH 8.0) at a final concentration of 0.1 mg/ml. The secondary structural characteristics of the proteins were estimated.

### Untargeted metabolomic analyses.

The mycelia of A226 and A226Δ*acrT* grown in R5 liquid medium for 72 h were harvested by centrifugation and washed at least three times with 50 mM phosphate buffer (pH 8.0). To remove proteins and obtain diverse metabolites, a mixture of methanol, acetonitrile, and water (2:2:1, vol/vol/vol) was added to the samples. After ultrasonic treatment, proteins were precipitated at −20°C for 1 h, followed by centrifugation (14,000 × *g*, 20 min, 4°C). The supernatant was used to monitor and balance the system for quality control (QC) purposes. Metabolomic analysis was performed using an LC-MS/MS system consisting of a model 1290 Infinity UPLC (Agilent), a model 6550 mass spectrometer (Agilent), and a TripleTOF 6600 mass spectrometer (AB SCIEX) at Shanghai Applied Protein Technology Co., Ltd. Based on the data library from Shanghai Applied Protein Technology Co., Ltd., MetaboAnalyst 5.0 (https://www.metaboanalyst.ca) was used for the multivariate statistical analysis, including principal-component analysis (PCA), partial least-squares discrimination analysis (PLS-DA), and orthogonal partial least-squares discrimination analysis (OPLS-DA).

### Detection of intracellular acyl-CoAs.

Based on a previously described method (29), the extraction and detection of various intracellular acyl-CoAs were performed with minor revisions. The mycelia of *Sac. erythraea* were separated from fermentation broths grown in R5 liquid medium for 72 h by centrifugation, washed at least three times with 50 mM phosphate buffer (pH 8.0), and lysed in buffer (10 mM DTT and 10% trichloroacetic acid). The lysates were frozen and thawed three times with liquid nitrogen and ice water. After centrifugation at 4°C, the supernatants were transferred to Sep-Pak C_18_ solid-phase extraction columns (Waters), washed with 0.1% trifluoroacetic acid (TFA), and eluted with 40% acetonitrile containing 0.1% TFA. The eluent was dried by a vacuum freeze dryer (Scientz). Acyl-CoAs were isolated and determined using an HPLC (Thermo Fisher Scientific), which was equipped with an InertSustain C_18_ column (5 μm, 4.6 by 250 mm; Shimadzu) and equilibrated with a mixture of 98% solution A (50 mM KH_2_PO_4_, pH 5.5) and 2% solution B (acetonitrile). Samples were detected at 254 nm with a flow rate of 0.8 ml/min. The mobile-phase compositions were set to several gradients of 0 to 8 min (solution A from 98% to 95%), 8 to 12 min (solution A from 95% to 90%), 12 to 15 min (solution A from 90% to 85%), 15 to 19 min (solution A from 85% to 70%), and 19 to 22 min (solution A from 70% to 98%). The column was equilibrated with the aforementioned mixture for 10 min.

### Statistical analysis.

All data are presented as means ± standard deviations (SDs) and were estimated by Student’s two-tailed *t* test. *P* values of less than 0.05 were considered statistically significant. Significance is indicated as *P < *0.05 (*), *P < *0.01 (**), and *P < *0.001 (***); ns indicates not significant. All error bars represent the SDs between independent experimental replicates.

### Data availability.

All data supporting the findings of this work are presented in the paper and the supplemental material.
