# Supplementary material for: Polyketide Starter and Extender Units Serve as Regulatory Ligands to Coordinate the Biosynthesis of Antibiotics in Actinomycetes
Source: mBio. 2021 Sep 28;12(5):e02298-21. doi: 10.1128/mBio.02298-21 (PMC8546615; doi:10.1128/mBio.02298-21)
Supplement: FIG S1 [file mbio.02298-21-sf001.pdf]

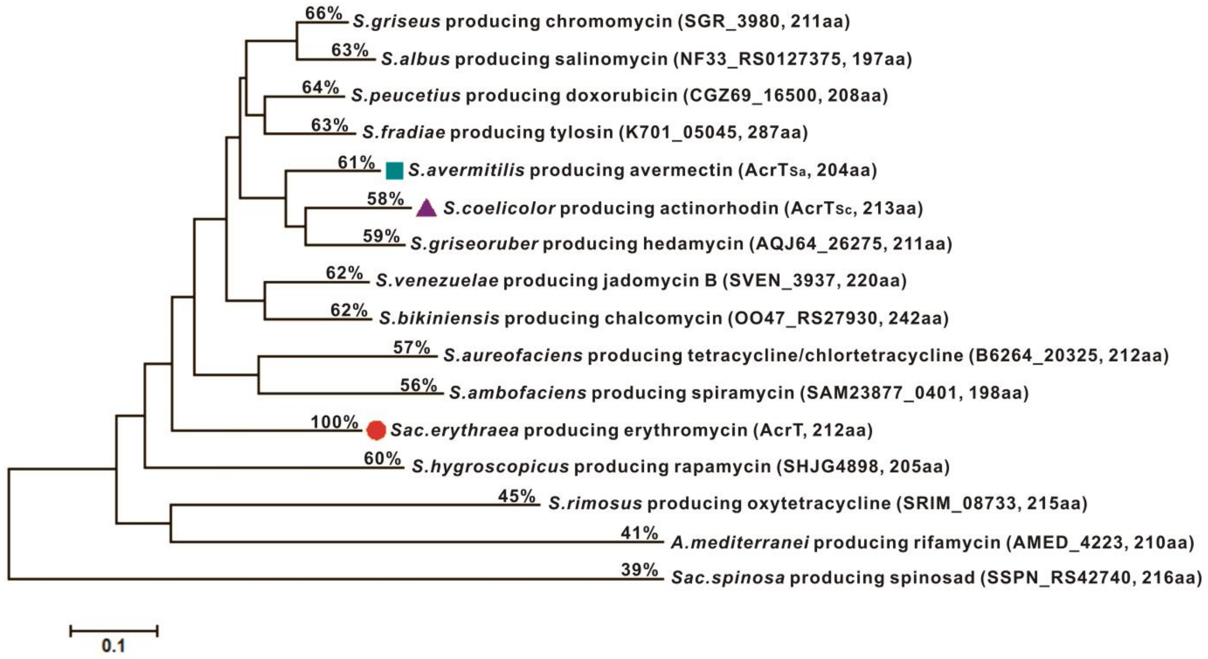


**FIG S1** The construction of neighbor-joining (NJ) distance tree of AcrT in *Sac.erythraea* and its homologs in polyketide-producing actinomycetes. The tree was constructed based on the amino acid sequences of AcrT and its homologs in polyketide-producing actinomycetes with MEGA (v6.06). Percentages represent the identities between AcrT and its homologs. Square, circle and triangle stand for the bacteria studied in this work.
