# Supplementary material for: Polyketide Starter and Extender Units Serve as Regulatory Ligands to Coordinate the Biosynthesis of Antibiotics in Actinomycetes
Source: mBio. 2021 Sep 28;12(5):e02298-21. doi: 10.1128/mBio.02298-21 (PMC8546615; doi:10.1128/mBio.02298-21)
Supplement: FIG S2 [file mbio.02298-21-sf002.pdf]

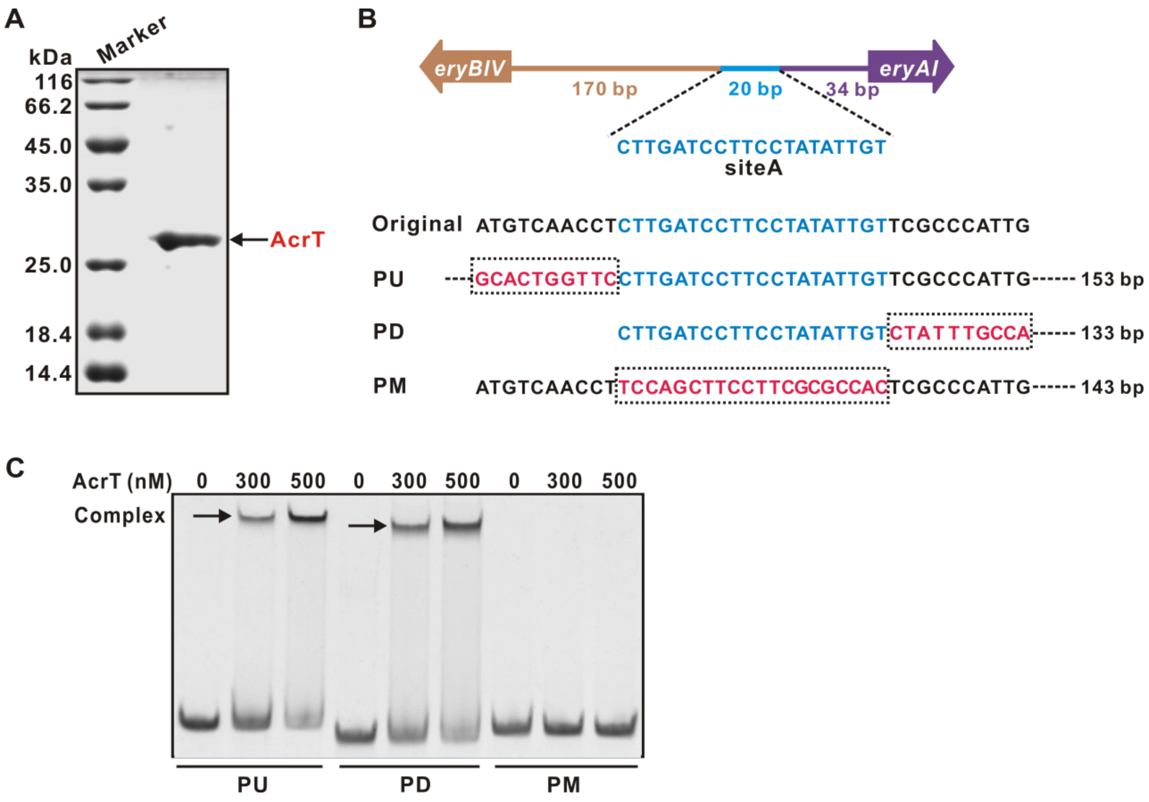

FIG S2 Determination of the precise site within P_{eryAI} for AcrT to bind. (A) Identification of His-tagged AcrT by SDS-PAGE. (B) Illustration of the mutated probes. PU probe, 10 adjacent bases upstream of site A were mutated; PD probe, 10 adjacent bases downstream of site A were mutated; PM probe, site A was mutated. The mutated bases are marked with dashed boxes. (C) EMSA of AcrT binding to the mutated probe PU, PD, or PM.
