# Supplementary material for: Polyketide Starter and Extender Units Serve as Regulatory Ligands to Coordinate the Biosynthesis of Antibiotics in Actinomycetes
Source: mBio. 2021 Sep 28;12(5):e02298-21. doi: 10.1128/mBio.02298-21 (PMC8546615; doi:10.1128/mBio.02298-21)
Supplement: FIG S3 [file mbio.02298-21-sf003.pdf]

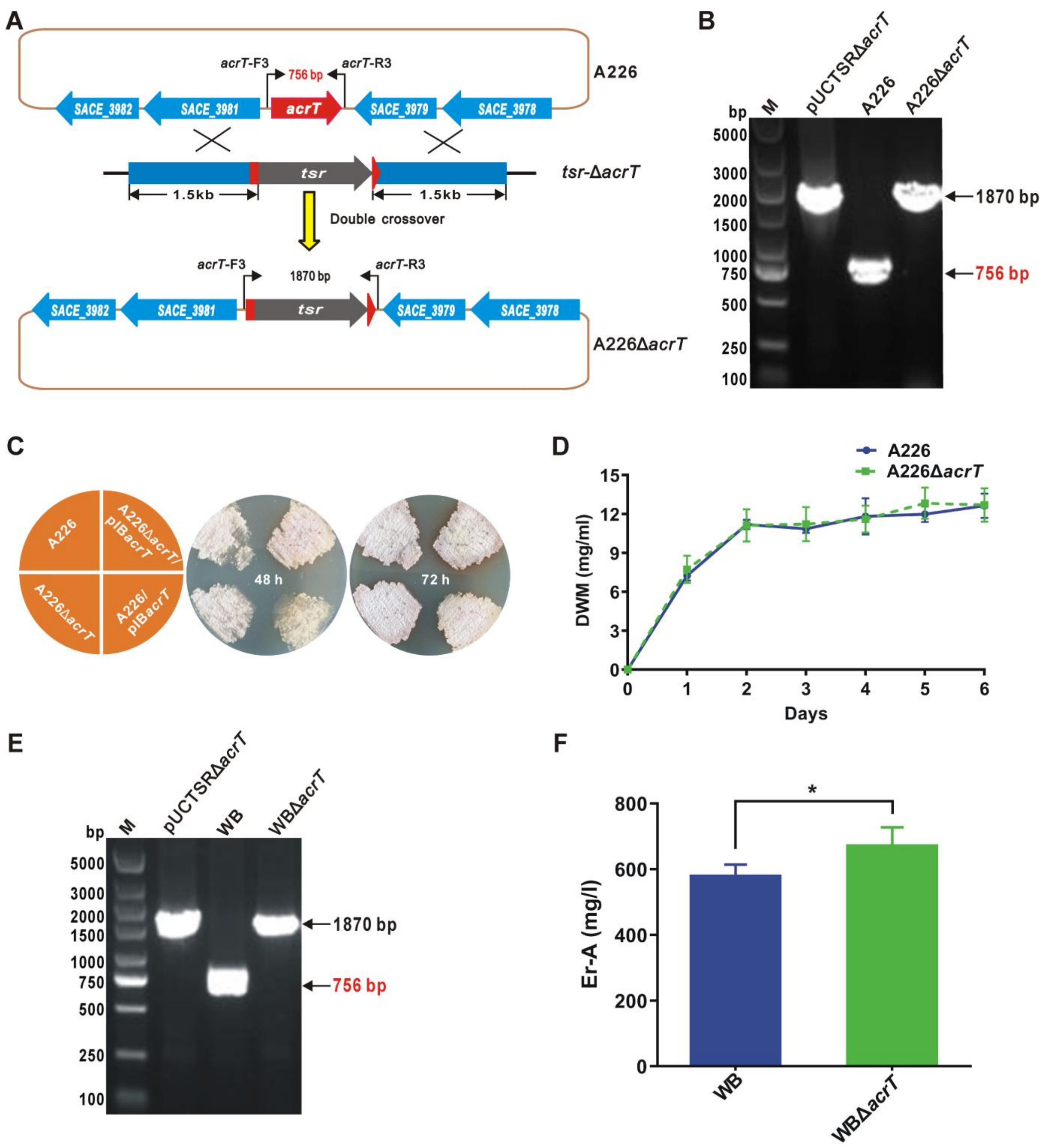

FIG S3 Inactivation of *acrT* in *Sac. erythraea*. (A) Schematic diagram of *acrT* deletion by homologous recombination with the linearized fragment in *Sac. erythraea* A226. (B) Identification of *acrT* deletion in *Sac. erythraea* A226. M, 5,000 bp DNA ladder; pUCTSRΔ*acrT*, the positive control, from which 1,870 bp DNA fragment was amplified; A226, the negative control, from which 756 bp DNA fragment was amplified; A226Δ*acrT*, the screened mutant, from which 1,870 bp DNA fragment was amplified. (C) Aerial mycelia formation of A226 and its derivatives. All strains were grown on R3M solid medium at

30 °C for 48 and 72 h. (D) Growth curves of A226 and A226Δ*acrT* in R5 liquid medium. Their dry weights of mycelia (DWM) were measured. (E) Confirmation of *acrT* deletion in the industrial strain *Sac. erythraea* WB. M, 5,000 bp DNA ladder; pUCTSRΔ*acrT*, the positive control, from which 1,870 bp DNA fragment was amplified; WB, the negative control, from which 756 bp DNA fragment was amplified; WBΔ*acrT*, the screened mutant, from which 1,870 bp DNA fragment was amplified. (F) Er-A production in WB and WBΔ*acrT*. Mean values of $n = 3$ measurements are shown with SDs. *, $P < 0.05$.
