# Supplementary material for: Polyketide Starter and Extender Units Serve as Regulatory Ligands to Coordinate the Biosynthesis of Antibiotics in Actinomycetes
Source: mBio. 2021 Sep 28;12(5):e02298-21. doi: 10.1128/mBio.02298-21 (PMC8546615; doi:10.1128/mBio.02298-21)
Supplement: TABLE S1 [file mbio.02298-21-st001.pdf]

Table S1. Strains, plasmids, and primers used in this study.

Materials	Descriptions	Sources or uses
Sac. erythraea		
A226	A low erythromycin producer	China Pharmaceutical Culture Collection, CGMCC 8279
A226Δ*acrT*	A226 with *acrT* deletion	This study
A226Δ*acrT*/pIB139	A226Δ*acrT* carrying pIB139	This study
A226Δ*acrT*/pIB*acrT*	A226Δ*acrT* carrying pIB139*acrT*	This study
A226/pIB139	A226 carrying pIB139	This study
A226/pIB*acrT*	A226 carrying pIB139*acrT*	This study
WB	An industrial erythromycin producer	China Pharmaceutical Culture Collection, CGMCC 8280
WBΔ*acrT*	WB with *acrT* deletion	This study
S. avermitilis		
NRRL8165	Avermectin producer (wild type)	China Pharmaceutical Culture Collection, CGMCC 4.3588
Δ*acrT*$_{Sa}$	NRRL 8165 with *acrT*$_{Sa}$ deletion	This study
Δ*acrT*$_{Sa}$/pIB139	Δ*acrT*$_{Sa}$ carrying pIB139	This study
Δ*acrT*$_{Sa}$/pIB*acrT*	Δ*acrT*$_{Sa}$ carrying pIB139*acrT*$_{Sa}$	This study
S. coelicolor		
M145	Actinorhodin producer (derived from *S. coelicolor* A3(2))	American Type Culture Collection, ATCC BAA-471
Δ*acrT*$_{Sc}$	M145 with *acrT*$_{Sc}$ deletion	This study
Δ*acrT*$_{Sc}$/pIB139	Δ*acrT*$_{Sc}$ carrying pIB139	This study
Δ*acrT*$_{Sc}$/pIB*acrT*$_{Sc}$	Δ*acrT*$_{Sc}$ carrying pIB*acrT*$_{Sc}$	This study
E. coli		
DH5α	F *recA lacZ*M15	Invitrogen
BL21(DE3)	F^{-} *ompT hsdS*$_B$ ($r_B^{-} m_B^{-}$) *gal dcm* (DE3)	Novagen
ET12567(pUZ8002)	*recF dam- dcm- hsdS cat Km*	(1)
**Plasmids**		
pUCTSR	pUC18 derivative containing a 1.36 kb fragment of a thiostrepton resistance cassette in the *Bam*HI/*Sma*I sites	(2)
pUCTSRΔ*acrT*	pUCTSR derivative for *acrT* deletion	This study

pKC1139	*ori* (pSG5), *aac(3)IV*, *lacZ*	(3)
pKCTSRΔ$acrT_{Sa}$	pKC1139 derivative for $acrT_{Sa}$ deletion	This study
pKCTSRΔ$acrT_{Sc}$	pKC1139 derivative for $acrT_{Sc}$ deletion	This study
pIB139	ΦC31 *attP-int* locus, *aac(3)IV*, $oriT_{RK2}$, P_{ermE*}	(4)
pIB*acrT*	pIB139 derivative for expression of *acrT*	This study
pIB$acrT_{Sa}$	pIB139 derivative for expression of $acrT_{Sa}$	This study
pIB$acrT_{Sc}$	pIB139 derivative for expression of $acrT_{Sc}$	This study
pKC-Lrp-DE	pIB139 derivative containing *egfp* gene	(2)
pKC-EE	derived from pKC1139, inserting P_{eryAI} and *egfp* in the *Hin*dIII/*Bam*HI sites, with *egfp* under P_{eryAI}	This study
pKC-*acrT*-EE	derived from pKC-EE, inserting *acrT* and $P_{aac(3)IV}$ in the *Eco*RV/*Eco*RI sites, with *egfp* under P_{eryAI} and *acrT* under $P_{aac(3)IV}$	This study
pKC-AE	derived from pKC1139, inserting P_{aveA1} and *egfp* in the *Hin*dIII/*Bam*HI sites, with *egfp* under P_{aveA1}	This study
pKC-$acrT_{Sa}$-AE	derived from pKC-AE, inserting $acrT_{Sa}$ and $P_{aac(3)IV}$ in the *Eco*RV/*Eco*RI sites, with *egfp* under P_{aveA1} and $acrT_{Sa}$ under $P_{aac(3)IV}$	This study
pKC-CE	derived from pKC1139, inserting $P_{actI\text{-}ORF1}$ and *egfp* in the *Hin*dIII/*Bam*HI sites, with *egfp* under $P_{actI\text{-}ORF1}$	This study
pKC-$acrT_{Sc}$-CE	derived from pKC-CE, inserting $acrT_{Sc}$ and $P_{aac(3)IV}$ in the *Eco*RV/*Eco*RI sites, with *egfp* under $P_{actI\text{-}ORF1}$ and $acrT_{Sc}$ under $P_{aac(3)IV}$	This study
pET28a	*kan*, P_{T7}, His-tag	Novagen
pET28a*acrT*	pET28a derivative carrying *acrT* in the *Nde*I/*Hin*dIII sites	This study
pET28a$acrT_{Sa}$	pET28a derivative carrying $acrT_{Sa}$ in the *Eco*RI/*Hin*dIII sites	This study
pET28a$acrT_{Sc}$	pET28a derivative carrying $acrT_{Sc}$ in the *Nde*I/*Hin*dIII sites	This study

Primers[a]

B-*eryAI*-F	GAATCACTGATCCCATTCACCGGAGCAT	DACA of the biotinylated
B-*eryAI*-R	AGCTTTGACAGGTCCGCCACGCG	P_{eryAI}
B-pUC-F	GAGCGGATAACAATTTCACACAGGAAACAG	DACA of the biotinylated
B-pUC-R	GCCAGGGTTTTCCCAGTCACGAC	negative control
acrT-F1	CCCAAGCTTGCGGTGTTCATCAGCGCGAT (*Hin*dIII)	Inactivation of *acrT*
acrT-R1	GCTCTAGAGCTCGACGAACAGCCGGATG (*Xba*I)	
acrT-F2	CGGGGTACCAGATCACCACCGTCCTGCG (*Kpn*I)	
acrT-R2	CCGGAATTCAGCCTCAACGTGCGGTTCA (*Eco*RI)	

acrT-F3	GCATGCCACAAAGGCTAACTCGGT	PCR confirmation of A226Δ*acrT* or WBΔ*acrT*
acrT-R3	AAGTCAGCACAGGCGTCCTCAGT	
acrT-F4	TTCCATATGATGGCGGTCATGAGCGAGCC (*Nde*I)	Complementation and overexpression of *acrT*
acrT-R4	TGCTCTAGATCAGCCGCAGCAGGCGGCC (*Xba*I)	
apr-F	GGAGTGCATATGGTGCAATACGAATGGCGAAAAG	PCR confirmation of strains integrated with pIB139
apr-R	CTCAAAGCTTCAGCCAATCGACTGGCGAGCG	
$acrT_{Sa}$-F1	CCCAAGCTTGCGAACACCTCCCCGACCAC (*Hin*dIII)	Inactivation of $acrT_{Sa}$
$acrT_{Sa}$-R1	TGCTCTAGAGATGGCGATGTCCGACACGG (*Xba*I)	
$acrT_{Sa}$-F2	CGGGGTACCGACGTCCACGCCGACGCCGT (*Kpn*I)	
$acrT_{Sa}$-R2	CCGGAATTCTGCGTTCGTCGTCGACGGCG (*Eco*RI)	
$acrT_{Sa}$-F3	CGCCATATGATGGGTGAGATCGGGCTGCG (*Nde*I)	PCR confirmation of $\Delta acrT_{Sa}$ and $\Delta acrT_{Sa}$/pIB$acrT_{Sa}$
$acrT_{Sa}$-R3	TGCTCTAGATCAGGCGTACGAGGGAAGCC (*Xba*I)	
$acrT_{Sc}$-F1	AAAGAATTCTGGCGGATGCCGGGTTCACCGACC (*Eco*RI)	Inactivation of $acrT_{Sc}$
$acrT_{Sc}$-R1	AAAGGTACCCTCGGCAGCGGCCGCCACCTCC (*Kpn*I)	
$acrT_{Sc}$-F2	AAATCTAGACAACGGGTCCTCGCCCTGGACAACTGG (*Xba*I)	
$acrT_{Sc}$-R2	AACAAGCTTACCAGCGCGCCGACCTCCACGA (*Hin*dIII)	
$acrT_{Sc}$-F3	AAACATATGGTGCGTAGGCTCGCCTGCATGA (*Nde*I)	PCR confirmation of $\Delta acrT_{Sc}$ and $\Delta acrT_{Sc}$/pIB$\Delta acrT_{Sc}$
$acrT_{Sc}$-R3	CGCTCTAGATTACATATTTTCTGTCGTAACAAATCTGGGCA (*Xba*I)	
EE-F1	AAAAAGCTTCGGAGCATTTGCTCGCTTTCCA (*Hin*dIII)	Construction of pKC-EE and pKC-*acrT*-EE
EE-R1	AAATCTAGAGCGTCCCCCTACTCGACGACCA (*Xba*I)	
EE-F2	AAATCTAGAATGGTGAGCAAGGGCGAGGAGCTG (*Xba*I)	
EE-R2	AAAGGATCCTTACTTGTACAGCTCGTCCATGCCGA (*Bam*HI)	
EE-F3	AAAGATATCGGGGTCTGACGCTCAGTGGAAC (*Eco*RV)	
EE-R3	AAACATATGCAGTCGATCATAGCACGATCAACG (*Nde*I)	
acrT-EE-F	AAACATATGATGGCGGTCATGAGCGAGCCGG (*Nde*I)	

acrT-EE-R	AAAGAATTCTCAGCCGCAGCAGGCGGCCA (*Eco*RI)	
AE-F1	AAAAAGCTTGGTCGGGAACCTCCGCAATC (*Hin*dIII)	Construction of pKC-AE and pKC-*acrT*$_{Sa}$-AE
AE-R1	ACCTCTAGACTGAGCTGTGTCCTCACCGCTA (*Xba*I)	
AE-F2	AAATCTAGAATGGTGAGCAAGGGCGAGGA (*Xba*I)	
AE-R2	AAAGGATCCTTACTTGTACAGCTCGTCCA (*Bam*HI)	
AE-F3	AAAGATATCGGGGTCTGACGCTCAGTGG (*Eco*RV)	
AE-R3	AAACATATGCAGTCGATCATAGCACGATCA (*Nde*I)	
acrT$_{Sa}$-AE-F	AAACATATGATGGGTGAGATCGGGCTGCG (*Nde*I)	
acrT$_{Sa}$-AE-R	AACGAATTCTCAGGCGTACGAGGGAAGCC (*Eco*RI)	
CE-F1	AAAAAGCTTACACGCGCAACCCCTCCTTAC (*Hin*dIII)	Construction of pKC-CE and pKC-*acrT*$_{Sc}$-CE
CE-R1	AAATCTAGACCCATCTCCCTTCGACCGCCG (*Xba*I)	
CE-F2	AAATCTAGAATGGTGAGCAAGGGCGAGGAGC (*Xba*I)	
CE-R2	AAAGGATCCTTACTTGTACAGCTCGTCCATGCCG (*Bam*HI)	
CE-F3	AAAGATATCGGGGTCTGACGCTCAGTGGAA (*Eco*RV)	
CE-R3	AAACATATGCAGTCGATCATAGCACGATCAA (*Nde*I)	
acrT$_{Sc}$-CE-F	AAACATATGGTGCGTAGGCTCGCCTGCATG (*Nde*I)	
acrT$_{Sc}$-CE-R	GGCGAATTCTTACATATTTTCTGTCGTAACAAATCTGGG (*Eco*RI)	
acrT-28a-F	CGCCATATGATGGCGGTCATGAGCGA (*Nde*I)	Expression of *acrT* in *E. coli*
acrT-28a-R	CCCAAGCTTTCAGCCGCAGCAGGC (*Hin*dIII)	
acrT$_{Sa}$-28a-F	ACCGAATTCATGGGTGAGATCGGGCTGCG (*Eco*RI)	Expression of *acrT*$_{Sa}$ in *E. coli*
acrT$_{Sa}$-28a-R	CCCAAGCTTTCAGGCGTACGAGGGAAGCC (*Hin*dIII)	
acrT$_{Sc}$-28a-F	AGCCATATGGTGCGTAGGCTCGCCTGC (*Nde*I)	Expression of *acrT*$_{Sc}$ in *E. coli*
acrT$_{Sc}$-28a-R	CCCAAGCTTTTACATATTTTCTGTCGTAACAAATCTGGG (*Hin*dIII)	
eryAI-BIV-F	CGGAGCATTTGCTCGCTTTCCAGG	EMSA analysis of P$_{eryAI}$
eryAI-BIV-R	GCGTCCCCCTACTCGACGACCAC	
ermE-CI-F	TGCTCGTCCGAACTGCTCACCGCT	EMSA analysis of *ermE-eryCI-int*
ermE-CI-R	TCCAGGAAGGGGACGTCCATGCGA	
eryBI-BIII-F	AGTCCCACAAGGAAGATCATAACGAGCGCGG	EMSA analysis of *eryBI-eryBIII-int*
eryBI-BIII-R	ACGCGCTCACCCCCAGTCATGCA	
eryBVI-F	TCGTCGGTGTGCGCGGATGACCC	EMSA analysis of P$_{eryBVI}$
eryBVI-R	GTCGCCGGTCCGATCACCCATCGA	
eryK-F	CACCGCGGAAGTCTCGACACCCCG	EMSA analysis of P$_{eryK}$

eryK-R	GCATCGTGCCCAGCCAGTCGAGGA	
acrT-3981-F	CGGTGGAGGCCGTCGTTCCGGTCAT	EMSA analysis of
acrT-3981-R	GACCCGGCTCGCTCATGACCGCCAT	*acrT-3981-int*
0018-0028-F	GACGTCCTCCTCCTGGTGCGGTCGGTTT	EMSA analysis of
0018-0028-R	ACTCACCACGTCCCTCCTCACAGGTCGTCG	$P_{0018-0028}$
0632-0633-F	GCTGCTTCTTTAGCACACCGCTACCAGCG	EMSA analysis of
0632-0633-R	GATGCCTCGTCTCTCACGCTCGGTGG	$P_{0632-0633}$
0649-F	GTCCCACCTGCCCAAAAGGCCAA	EMSA analysis of P_{0649}
0649-R	TCGGGAACCTCTCGCGCTCGTCA	
3241-3242-F	GGAGATCCCGGAGGTGTTGCTGTG	EMSA analysis of
3241-3242-R	TGGTGTCCACTTGCGACTTCAGCG	$P_{3241-3242}$
3398-3399-F	AGGCCCATCGGTCCGCCCGCTGCTG	EMSA analysis of
3398-3399-R	TCCGAACCCGCGTGCACGGCCTCG	$P_{3398-3399}$
3400-F	TCCCCTGTAAAGCAATTCCCCGCGCGAG	EMSA analysis of P_{3400}
3400-R	CCGCATCCACATCCGGCTCCGCATACA	
4237-F	ACTGCGCGTGGCAGCAACCTGCCAGG	EMSA analysis of P_{4237}
4237-R	GGCAACACGACTGAACACCGACTCGAGCCT	
7038-7039-F	CGCGTAGGACTCCGAGGCCGGGTCG	EMSA analysis of
7038-7039-R	CTTCCAGGATCTGCTCGCGCCGGG	$P_{7038-7039}$
FAM-*eryAI-BIV*-F	CGGAGCATTTGCTCGCTTTCCAGG	DNase I footprinting
HEX-*eryAI-BIV*-R	GCGTCCCCCTACTCGACGACCAC	assay of *eryAI-eryBIV-int*
U-*eryAI*-F	CGACGAGGCGGCACTGGTTCCTTGATCCTTCC TATATTGT	EMSA analysis of the probes PU, PM, and PD
M-*eryAI*-F	ATGTCAACCTTCCAGCTTCCTTCGCGCCACTCG CCCATTGCGTGGTCGTC	
D-*eryAI*-F	CTTGATCCTTCCTATATTGTCTATTTGCCACGTG GTCGTCGAGTAGGGGG	
O-*eryAI*-R	ACCCCGACAGGGGCCACGGACGA	
BLI-*eryAI*-F1	CGGAGCATTTGCTCGCTTTCCAGG	The biotinylated P_{eryAI}
BLI-*eryAI*-R1	GCGTCCCCCTACTCGACGACCAC	used for BLI
BLI-*eryAI*-F2	AGGCGATGTCAACCTCTTGATCCTTCCTATATT GTTCGCCCATTGCGTGG	The 50 bp biotinylated probe containing siteA
BLI-*eryAI*-R2	CCACGCAATGGGCGAACAATATAGGAAGGATC AAGAGGTTGACATCGCCT	used for BLI
aveA1-F	TCGGGAACCTCCGCAATCCG	EMSA analysis of P_{aveA1}
aveA1-R	CTGAGCTGTGTCCTCACCGCTAGG	
actI-ORF1-F	CCGCTGGTCGCACCCGTCACCAGTG	EMSA analysis of
actI-ORF1-R	GATACGGGACCCCTCGATGCCGGATCCA	$P_{actI-ORF1}$

Co-*0018-0019*-F	ACTTCCACAGCCGCCGGATGGGTCC	Identification of co-transcription of *SACE_0018-0026*
Co-*0018-0019*-R	TCCACGTCGACCTTGGCCGGCTCG	
Co-*0019-0020*-F	ACCGAGGCGCTGGTGCTCTCCGG	
Co-*0019-0020*-R	AGGTGTCGCCGGAGAGCTCCGCCG	
Co-*0020-0021*-F	ACGTTGGACTGCCCCGGCGACCA	
Co-*0020-0021*-R	ATGCAGTCCCGCGCCTCGACGACG	
Co-*0021-0022*-F	AGCGGTTCCCGGCGATGCCCGAACC	
Co-*0021-0022*-R	ACAGAGCATCGGCGAGCGCGGTGC	
Co-*0022-0023*-F	AGGGCTCGGTGCGGGCCACCGC	
Co-*0022-0023*-R	TCGCGACCACCGTGGCTGCGCTG	
Co-*0023-0024*-F	ACGACGACCGGGTGGTCGTGGTGGC	
Co-*0023-0024*-R	GACGACGTAGCAGGGCCGGTCGAGCC	
Co-*0024-0025*-F	AGGTCGCCTGGCAACTGCTGCGCGG	
Co-*0024-0025*-R	ATTCGTCGCGCCCGCGCATCGCG	
Co-*0025-0026*-F	GCACGTGAGGGCGCTGCACGGCTC	
Co-*0025-0026*-R	TGTGGTGCGCGCAGTCCGGGCAGA	
Co-*0632-0633*-F	TCACCGCCCGCGTGATCGCCTCC	Identification of co-transcription of *SACE_0632-0633*
Co-*0632-0633*-R	GGTCCTCCGCCCGGTAGACCCTGTGGC	
RT-*acrT*-F1	AAGGAGGACCTGGTACTGCAC	Detection of relative transcription of *acrT*
RT-*acrT*-R1	GTAGACCAGTCCGCTGAAGG	
RT-*acrT*-F2	ATGGCGGTCATGAGCGAGCC	RT-qPCR analysis of *acrT*
RT-*acrT*-R2	GCTCGACGAACAGCCGGAT	
RT-*eryAI*-F	GACCTGTCAAAGCTCTCCGA	RT-qPCR analysis of *eryAI*
RT-*eryAI*-R	GATCGAGATGTGCACGCAAT	
RT-*eryBIV*-F	GGTCAACGTCGGCCTGAT	RT-qPCR analysis of *eryBIV*
RT-*eryBIV*-R	GTCTTCTGCTGCGCGTAC	
RT-*ermE*-F	GAGTGGGAGTTCGTCGAGA	RT-qPCR analysis of *ermE*
RT-*ermE*-R	ACCATCGACTCGTAGCGTTC	
RT-*eryCI*-F	TTCTACCCGGGCAAGAACC	RT-qPCR analysis of *eryCI*
RT-*eryCI*-R	GCACGTACTTCTGCTTCGAG	
RT-*eryBI*-F	CACTACTCGGAGGGCATCTT	RT-qPCR analysis of *eryBI*
RT-*eryBI*-R	CGAACGTGGTGTAGGAAAGC	
RT-*eryBIII*-F	ACGTCATCCTCCAGTACTGC	RT-qPCR analysis of *eryBIII*
RT-*eryBIII*-R	CCTCTTCCTCCGACACGATC	
RT-*eryBVI*-F	AACCGGTACATGCTGATCGA	RT-qPCR analysis of *eryBVI*
RT-*eryBVI*-R	GCAGCTGGATGTTGACGTAG	

RT-*eryK*-F	GATCGCGGATGTGCTCAAC	RT-qPCR analysis of *eryK*
RT-*eryK*-R	CAGGACAAGGCGGGAGATC	
RT-*0026-0028*-F	GTGTCCTGGTCGTCAGCAT	RT-qPCR analysis of *SACE_0026-0028*
RT-*0026-0028*-R	CAACTCCTGCCTGATGGTCT	
RT-*3241-3242*-F	GCCTCAACTGGACCAAGAAG	RT-qPCR analysis of *SACE_3241-3242*
RT-*3241-3242*-R	GGCTAGGACCGTAGGAGGTC	
RT-*3398-3399*-F	TGTTCTCCCAGGACTTCACC	RT-qPCR analysis of *SACE_3398-3399*
RT-*3398-3399*-R	GCTTGAAGATCTCGGCGTAG	
RT-*3400*-F	GAGGCGATGAAGATGGAGAA	RT-qPCR analysis of *SACE_3400*
RT-*3400*-R	TCAGTCCTTGAGTTCGCAGA	
RT-*3856*-F	GTTCGACTTCGGCTTCCTC	RT-qPCR analysis of *SACE_3856*
RT-*3856*-R	CAGGATTCCCTCCTGCATC	
RT-*4237*-F	TGGTCGCAGACATCTACCTG	RT-qPCR analysis of *SACE_4237*
RT-*4237*-R	CTCGAAGGTGAAGTGGTGGT	
RT-*6509*-F	GAGGCGATGAAGATGGAGAA	RT-qPCR analysis of *SACE_6509*
RT-*6509*-R	TCAGTCCTTGAGTTCGCAGA	
RT-*7038-7039*-F	ACTACCCGATGACGGTCAAG	RT-qPCR analysis of *SACE_7038-7039*
RT-*7038-7039*-R	GGTCGCCTGGTTGTAGAAGA	
RT-*0632-0633*-F	GAGCAGTTCCTCACCCTCTG	RT-qPCR analysis of *SACE_0632-0633*
RT-*0632-0633*-R	GAACGTCGAGGCGTTCAG	
RT-*0649*-F	AACCAGGTCGAACTGTCCAC	RT-qPCR analysis of *SACE_0649*
RT-*0649*-R	GTCATCCGCAGGAAGTTCTC	
RT-*hrdB*-F1	CGATACGGGCAGACTTGA	RT-qPCR ananlysis of *hrdB* in *Sac. erythraea* as an internal control
RT-*hrdB*-R1	CGTTTACGGCGTGGACTA	
RT-*aveA1*-F	CAACGACTCCTCCTCGAAAC	RT-qPCR analysis of *aveA1*
RT-*aveA1*-R	AGATACGACCGGAGATGACG	
RT-*hrdB*-F2	TGACCCCTGAGAAGGTCATC	RT-qPCR ananlysis of *hrdB* in *S. avermitilis* as an internal control
RT-*hrdB*-R2	CTCGATGAGGTCACCGAACT	
RT-*actI-ORF1*-F	AAGGAGCTGTTCGGATTGAA	RT-qPCR analysis of *actI-ORF1*
RT-*actI-ORF1*-R	GAGGTGAGCAGTTCCCAGAA	
RT-*hrdB*-F3	TGGTCGAGGTCATCAACAAG	RT-qPCR ananlysis of *hrdB* in *S. coelicolor* as an internal control
RT-*hrdB*-R3	TGGACCTCGATGACCTTCTC	

[a]Primers are shown from 5′ to 3′ and underlined sequences represent restriction sites.

Supplemental References

1. Green MR, Sambrook J. 2012. Molecular cloning: a laboratory manual, 4th ed. Cold Spring Harbor Laboratory, Cold Spring Harbor, NY.
2. Liu J, Chen Y, Wang W, Ren M, Wu P, Wang Y, Li C, Zhang L, Wu H, Weaver DT, Zhang B. 2017. Engineering of an Lrp family regulator SACE_Lrp improves erythromycin production in *Saccharopolyspora erythraea*. Metab Eng 39:29–37. https://doi.org/10.1016/j.ymben.2016.10.012.
3. Bierman M, Logan R, O'Brien K, Seno ET, Rao RN, Schoner BE. 1992. Plasmid cloning vectors for the conjugal transfer of DNA from *Escherichia coli* to *Streptomyces* spp. *Gene* 116:43–49. https://doi.org/10.1016/0378-1119(92)90627-2.
4. Xu Y, Ke M, Li J, Tang Y, Wang N, Tan G, Wang Y, Liu R, Bai L, Zhang L, Wu H, Zhang B. 2019. TetR-type regulator SLCG_2919 is a negative regulator of lincomycin biosynthesis in *Streptomyces lincolnensis*. Appl Environ Microbiol 85:e02091–18. https://doi.org/10.1128/AEM.02091-18.
