# Supplementary material for: Polyketide Starter and Extender Units Serve as Regulatory Ligands to Coordinate the Biosynthesis of Antibiotics in Actinomycetes
Source: mBio. 2021 Sep 28;12(5):e02298-21. doi: 10.1128/mBio.02298-21 (PMC8546615; doi:10.1128/mBio.02298-21)
Supplement: FIG S4 [file mbio.02298-21-sf004.pdf]

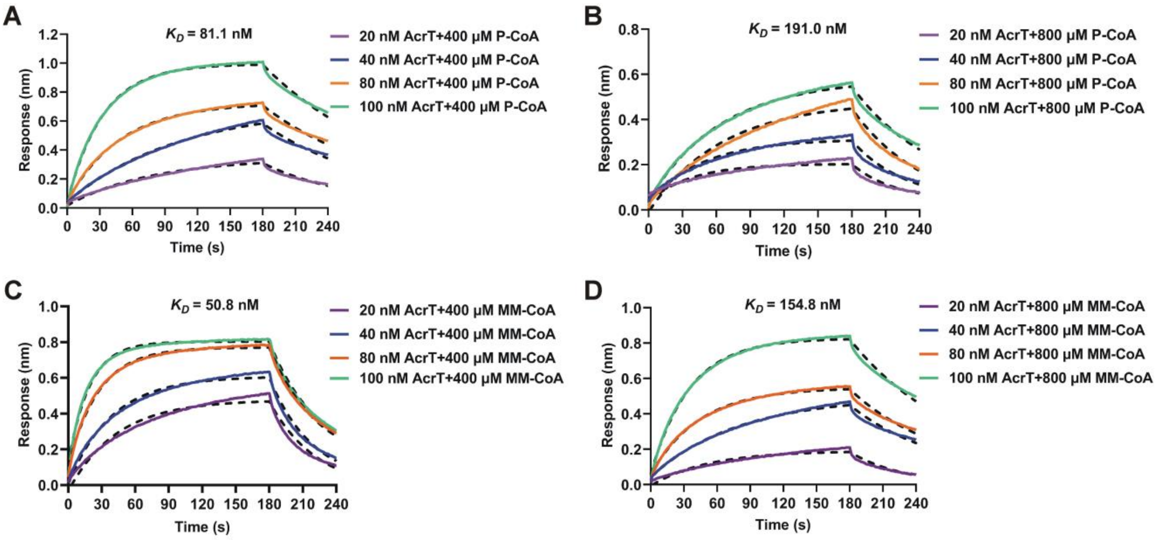

FIG S4 Affinity constant (K_D) analysis by BLI. (A) The affinity with the addition of 400 μM P-CoA. (B) The affinity with the addition of 800 μM P-CoA. (C) The affinity with the addition of 400 μM MM-CoA. (D) The affinity with the addition of 800 μM MM-CoA. The chart shows the binding curves for the 50 bp probe within P_{eryAI} against AcrT with different concentrations.
