# Supplementary material for: Polyketide Starter and Extender Units Serve as Regulatory Ligands to Coordinate the Biosynthesis of Antibiotics in Actinomycetes
Source: mBio. 2021 Sep 28;12(5):e02298-21. doi: 10.1128/mBio.02298-21 (PMC8546615; doi:10.1128/mBio.02298-21)
Supplement: FIG S5 [file mbio.02298-21-sf005.pdf]

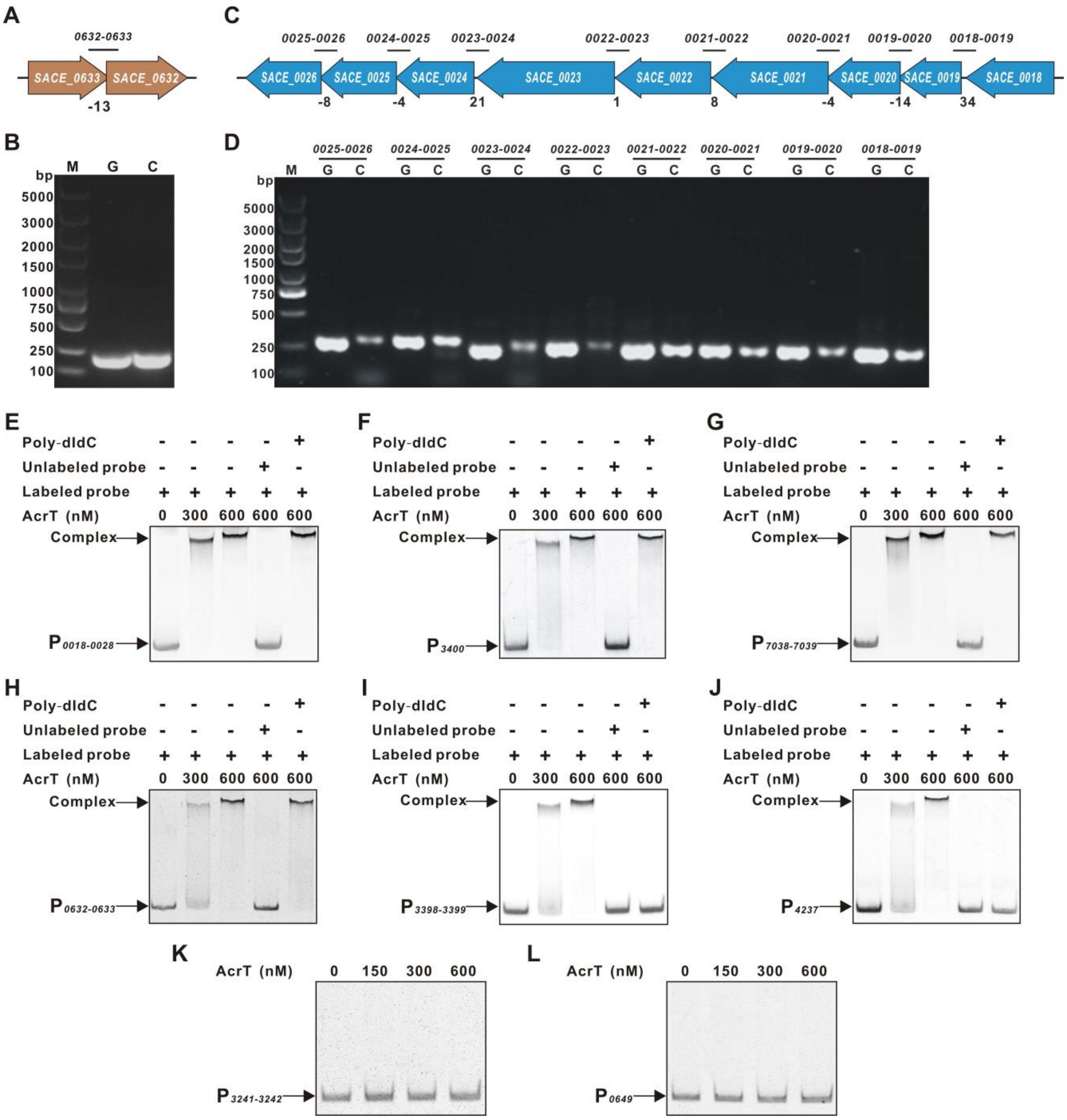

FIG S5 Interactions of AcrT with the promoters of ACC/PCC and CS genes. (A) PCR primer design of *SACE_0632-0633* (CS genes) for identifying the transcriptional unit. Solid line indicates the DNA fragment across *SACE_0632* and *SACE_0633* in A226. The negative number represents the overlapping region of these two genes. (B) Determination of transcriptional unit of *SACE_0632-0633*. Lane M, 5,000 bp DNA ladder; lane G, the PCR products using genomic DNA of A226 as the template; lane C, the PCR products using cDNA library of A226 as the template. (C) PCR primer design of *SACE_0018-0026* genes for identifying the transcriptional unit. Solid lines indicate DNA fragments across the adjacent genes in A226. A negative number represents overlapping region of two adjacent

genes, and a positive number represents intergenic region of two adjacent genes. (D) Determination of co-transcription of *SACE_0018-0026* genes. Here we determined that the real promoter of *SACE_0026-0028* genes was located upstream of *SACE_0018*, not upstream of *SACE_0026*. Lane M, 5,000 bp DNA ladder; lane G, the PCR products using genomic DNA of A226 as the template; lane C, the PCR products using cDNA library of A226 as the template. (E) EMSA with AcrT binding to $P_{0018-0028}$ (*SACE_0026-0028*, ACC genes). (F) EMSA with AcrT binding to P_{3400} (*SACE_3400*, ACC or PCC gene). (G) EMSA with AcrT binding to $P_{7038-7039}$ (*SACE_7038-7039*, ACC and/or PCC genes). (H) EMSA with AcrT binding to $P_{0632-0633}$ (*SACE_0632-0633*, CS genes). (I) EMSA with AcrT binding to $P_{3398-3399}$ (*SACE_3398-3399*, ACC and PCC genes). (J) EMSA with AcrT binding to P_{4237} (*SACE_4237*, ACC or PCC gene). (K) EMSA with AcrT binding to $P_{3241-3242}$ (*SACE_3241-3242*, ACC and/or PCC genes). (L) EMSA with AcrT binding to P_{0649} (*SACE_0649*, CS gene). $P_{3241-3242}$ and P_{0649} were used as the negative controls. Competing assays were performed using 50-fold excessive unlabeled probes or 50-fold excessive nonspecific probe poly-dIdC.
