# Supplementary material for: Polyketide Starter and Extender Units Serve as Regulatory Ligands to Coordinate the Biosynthesis of Antibiotics in Actinomycetes
Source: mBio. 2021 Sep 28;12(5):e02298-21. doi: 10.1128/mBio.02298-21 (PMC8546615; doi:10.1128/mBio.02298-21)
Supplement: FIG S6 [file mbio.02298-21-sf006.pdf]

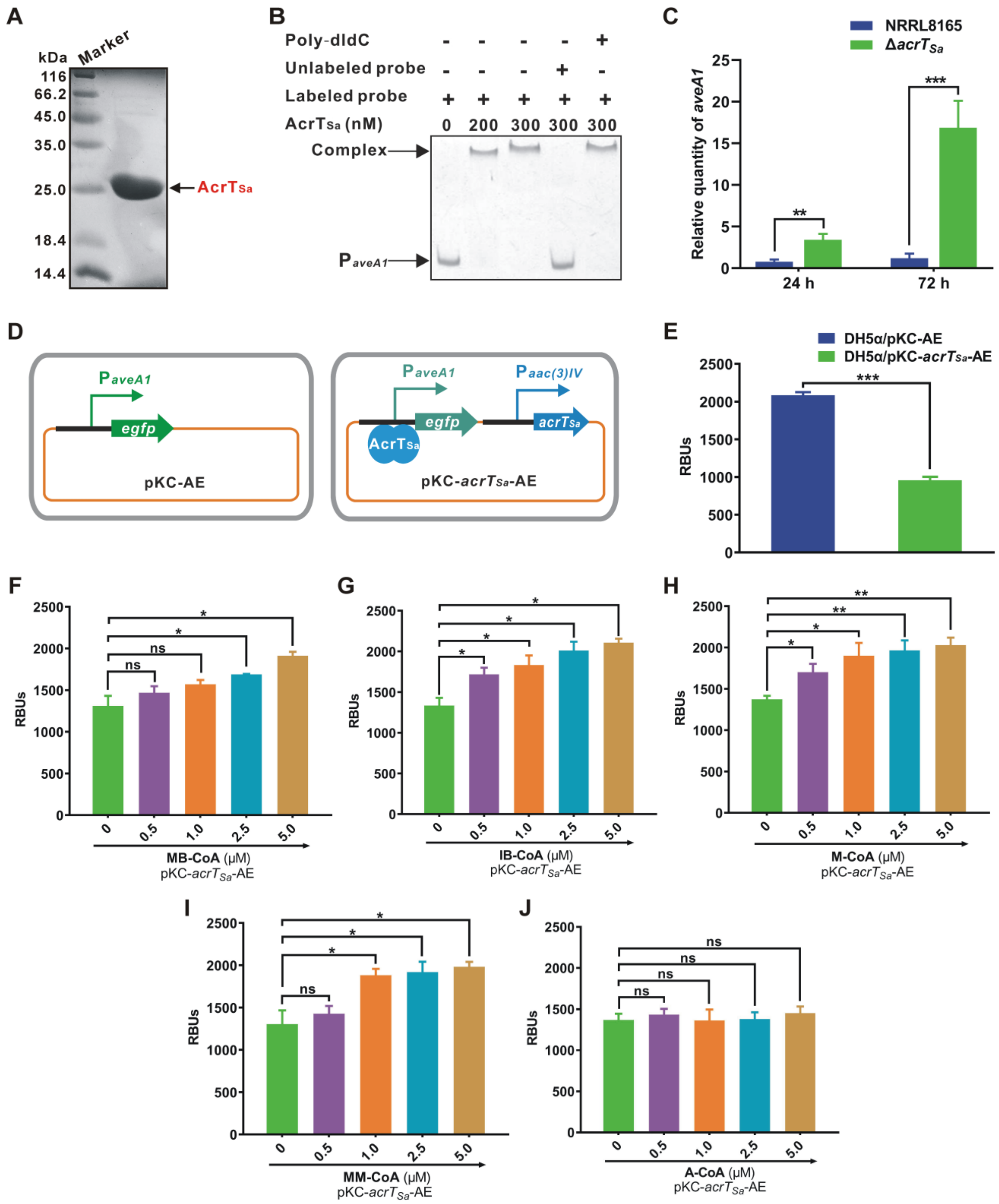

FIG S6 The starter and extender units relieve the repression of AcrT$_{Sa}$ on P$_{aveA1}$. (A) Identification of His-tagged AcrT$_{Sa}$ by SDS-PAGE. (B) EMSA of AcrT$_{Sa}$ binding to P$_{aveA1}$. Competing assays were performed using 50-fold excessive unlabeled P$_{aveA1}$ or 50-fold excessive nonspecific probe poly-dIdC. (C) RT-qPCR analyses of *aveA1* in *S.avermitilis* NRRL8165 and Δ*acrT$_{Sa}$* cultured for 24 and 72 h. (D) Illustration of the EGFP reporter system. The system used two plasmids, pKC-AE expressing *egfp*

under P_{aveA1} without $acrT_{Sa}$ and pKC-$acrT_{Sa}$-AE expressing *egfp* under P_{aveA1} with $acrT_{Sa}$ driven by $P_{aac(3)IV}$. (E) Detection of RBUs of the EGFP reporter system in *E. coli* DH5α. (F) Detection of RBUs in *E. coli* DH5α/pKC-$acrT_{Sa}$-AE with MB-CoA. (G) Detection of RBUs in *E. coli* DH5α/pKC-$acrT_{Sa}$-AE with IB-CoA. (H) Detection of RBUs in *E. coli* DH5α/pKC-$acrT_{Sa}$-AE with M-CoA. (I) Detection of RBUs in *E. coli* DH5α/pKC-$acrT_{Sa}$-AE with MM-CoA. (J) Detection of RBUs in *E. coli* DH5α/pKC-$acrT_{Sa}$-AE with A-CoA (the control). Mean values of $n = 3$ measurements are shown with SDs. *, $P < 0.05$; **, $P < 0.01$; ***, $P < 0.001$; ns, not significant.
