# Supplementary material for: Polyketide Starter and Extender Units Serve as Regulatory Ligands to Coordinate the Biosynthesis of Antibiotics in Actinomycetes
Source: mBio. 2021 Sep 28;12(5):e02298-21. doi: 10.1128/mBio.02298-21 (PMC8546615; doi:10.1128/mBio.02298-21)
Supplement: FIG S7 [file mbio.02298-21-sf007.pdf]

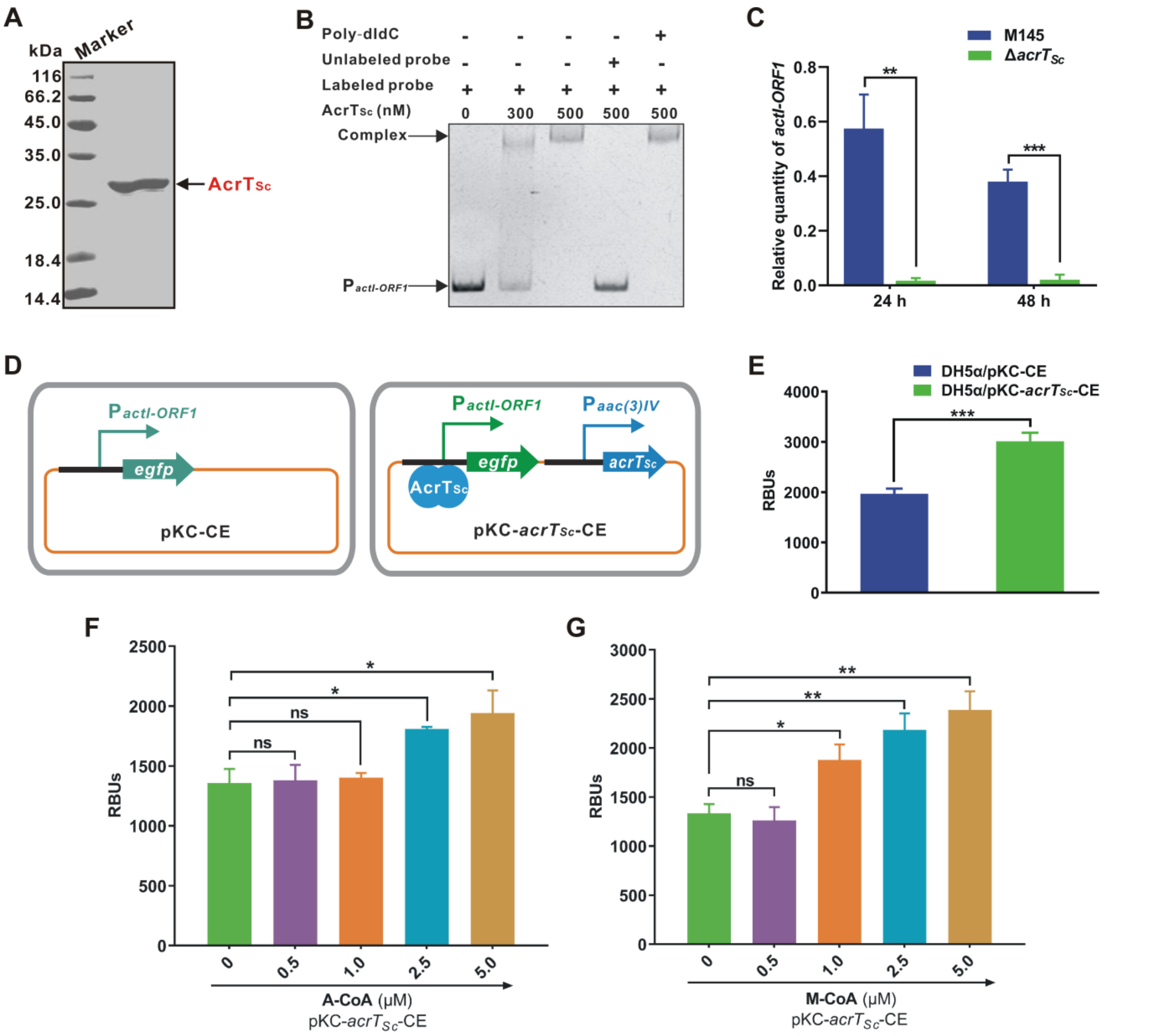

FIG S7 The starter and extender units promote the activation of $AcrT_{Sc}$ on $P_{actI\text{-}ORF1}$. (A) Identification of His-tagged $AcrT_{Sc}$ by SDS-PAGE. (B) EMSA of $AcrT_{Sc}$ binding to $P_{actI\text{-}ORF1}$. Competing assays were performed using 50-fold excessive unlabeled $P_{actI\text{-}ORF1}$ or 50-fold excessive nonspecific probe poly-dIdC. (C) RT-qPCR analyses of *actI-ORF1* in *S. coelicolor* M145 and Δ$acrT_{Sc}$ cultured for 24 and 48 h. (D) Illustration of the EGFP reporter system. The system used two plasmids, pKC-CE expressing *egfp* under $P_{actI\text{-}ORF1}$ without $acrT_{Sc}$ and pKC-$acrT_{Sc}$-CE expressing *egfp* under $P_{actI\text{-}ORF1}$ with $acrT_{Sc}$ driven by $P_{aac(3)IV}$. (E) Detection of RBUs of the EGFP reporter system in *E. coli* DH5α. (F) Detection of RBUs in *E. coli* DH5α/pKC-$acrT_{Sc}$-CE with A-CoA. (G) Detection of RBUs in *E. coli* DH5α/pKC-$acrT_{Sc}$-CE with M-CoA. Mean values of $n = 3$ measurements are shown with SDs. *, $P < 0.05$; **, $P < 0.01$; ***, $P < 0.001$; ns, not significant.
